# Investigating the global genomic diversity of *Escherichia coli *using a multi-genome DNA microarray platform with novel gene prediction strategies

**DOI:** 10.1186/1471-2164-12-349

**Published:** 2011-07-06

**Authors:** Scott A Jackson, Isha R Patel, Tammy Barnaba, Joseph E LeClerc, Thomas A Cebula

**Affiliations:** 1 Division of Molecular Biology, Office of Applied Research and Safety Assessment, Center for Food Safety and Applied Nutrition, U.S. Food and Drug Administration, Laurel, Maryland 20708, USA; 2 Department of Biology, Johns Hopkins University, 3400 N. Charles St., Baltimore, MD 21218, USA

**Keywords:** genome, diversity, microarray, *Escherichia coli*, *Shigella*, O157:H7, gene content, pathogenic determinants

## Abstract

**Background:**

The gene content of a diverse group of 183 unique *Escherichia coli *and *Shigella *isolates was determined using the Affymetrix GeneChip^® ^*E. coli *Genome 2.0 Array, originally designed for transcriptome analysis, as a genotyping tool. The probe set design utilized by this array provided the opportunity to determine the gene content of each strain very accurately and reliably. This array constitutes 10,112 independent genes representing four individual *E. coli *genomes, therefore providing the ability to survey genes of several different pathogen types. The entire ECOR collection, 80 EHEC-like isolates, and a diverse set of isolates from our FDA strain repository were included in our analysis.

**Results:**

From this study we were able to define sets of genes that correspond to, and therefore define, the EHEC pathogen type. Furthermore, our sampling of 63 unique strains of O157:H7 showed the ability of this array to discriminate between closely related strains. We found that individual strains of O157:H7 differed, on average, by 197 probe sets. Finally, we describe an analysis method that utilizes the power of the probe sets to determine accurately the presence/absence of each gene represented on this array.

**Conclusions:**

These elements provide insights into understanding the microbial diversity that exists within extant *E. coli *populations. Moreover, these data demonstrate that this novel microarray-based analysis is a powerful tool in the field of molecular epidemiology and the newly emerging field of microbial forensics.

## Background

*Escherichia coli *is a Gram negative bacterium that is commonly found in the lower intestine of warm-blooded organisms. Most *E. coli *strains are harmless. As such, commensal strains of *E. coli *are part of the normal microbiota of the lower gastrointestinal tract (GI), benefitting their hosts by producing vitamin K2 and also by preventing the establishment of pathogenic bacteria within the intestine through a "colonization barrier effect". Yet some strains can cause infections in humans. Six major categories of diarrheagenic *E. coli *exist: enterotoxigenic *E. coli *(ETEC), enteroinvasive *E. coli *(EIEC), enteropathogenic *E. coli *(EPEC), enterohemorrhagic *E. coli *(EHEC), enteroaggregative *E. coli *(EAEC), and diffusely adherent *E. coli *(DAEC). Furthermore various types of extraintestinal pathogenic *E. coli *(ExPEC) are known to cause infections outside the gastrointestinal tract; namely uropathogenic *E. coli *(UPEC) and newborn meningitis-associated *E. coli *(NMEC).

*E. coli *O157:H7, an EHEC pathotype, was first recognized as a human pathogen in 1982 [1]. It has emerged as a major enteric pathogen capable of causing outbreaks of food poisoning in humans and often responsible for costly food product recalls. The primary clinical manifestation of an *E. coli *O157:H7 infection is hematic diarrhea that can progress into more severe sequelae of hemolytic-uremic syndrome and thrombocytopenic thrombotic purpura, which then can lead to renal failure and death [2-4]. An estimated 75,000 cases of *E. coli *O157:H7 infections occur annually in the United States, making it the principal serotype of enterohemorrhagic *E. coli *isolated from patients [5]. Infections with this serotype usually have a food borne etiology. In 2006, for example, three multi-state and 24 single-state foodborne outbreaks were confirmed to be due to *E. coli *O157:H7 [6].

The extent of genomic diversity existing in *E. coli *and other microbial pathogen populations has been the subject of debate. Recent studies including several genomic methods like DNA microarrays, optical mapping, and whole genome sequencing have shed new light on the level of diversity within bacterial populations, making it clear that the level of genomic diversity had been grossly underestimated previously [7-9]. A comprehensive sampling of this diverse species, however, has not been addressed in any single study using an advanced whole-genome analysis method such as microarray analysis.

Previous microarray studies have been limited to sampling a relatively small and/or undiverse collection of *E. coli *strains. Moreover, traditional microarrays used in these studies have had limited gene content and typically utilize a single probe for determining whether a gene is present or absent within a given strain [10]. This makes gene detection calls questionable and absolutely requires the inclusion of a reference strain for making accurate gene calls.

Current array technology allows the design of microarrays containing greater than six million features. Arrays thus can be designed to probe the genomes of multiple organisms, a prerequisite of great value to an investigator who may study closely related strains or species. However, a caveat in multiple genome array design is that it is not often possible, nor desirable, to define unique probes that match every gene target with 100% sequence homology. It is therefore necessary to establish a consensus sequence for allelic variants. When a consensus sequence is represented on the array, consideration of the level of sequence homology of a particular target sequence is important to accurately determine the status of that gene following a hybridization experiment.

The Affymetrix GeneChip^® ^*E. coli *Genome 2.0 Array represents the genic and intergenic sequences of four sequenced strains of *E. coli*: enterohemorrhagic (EHEC) O157:H7 strains EDL933 and Sakai, the uropathogenic (UPEC) O6:H1:K2 strain CFT073, and the laboratory attenuated K12 strain MG1655 (OR:H48:K-) [11]. The array consists of 228,484 25-mer oligonucleotides that represent a total of 10,208 probe sets. Each probe set contains approximately 22 oligonucleotide probes; 11 perfect match (PM) probes and 11 mismatch probes (MM). Mismatch probes are identical to the perfect match probe with the exception of a one nucleotide (nt) mismatch located at the 13th (middle) position of the oligo sequence. These mismatch probes are designed to allow for an approximation, and correction, of non-specific hybridization signal. We presumed that a probe design strategy such as this would be ideally suited for genotyping studies for two reasons: i) the probe redundancy for each genomic target sequence and ii) the excellent specificity afforded by hybridization of short 25-mer probes. Previous microarray studies have used either long oligos (50-mers to 80-mers) or PCR-derived cDNA amplicons. Inherent to these array designs are the disadvantages that i) only a single probe signal is used to measure the presence of each genomic target and ii) relatively high non-specific hybridization signal is observed when using longer DNA probes [12,13].

In this study, we used the Affymetrix GeneChip^® ^*E. coli *Genome 2.0 Array to investigate the gene content of 207 diverse isolates of *E. coli *and *Shigella*. This multi-genome array provided us the opportunity to survey genes from different pathogen groups [11]. Strains of *E. coli *and *Shigella *interrogated in this study consist of approximately 60 different serotypes and 75 isolates of the O157:H7 serotype (Table 1). Moreover, the entire ECOR collection [14] was included in this study and contains a set of 72 reference strains isolated from a variety of hosts and geographical locations that is presumed to represent the range of phenotypic and genotypic variation in the *E. coli *species as a whole. Finally, four sequenced strains of *Shigella*, believed to be in the same species division as *E. coli*, were included in this study. In summary, this strain collection was chosen both to i) represent the global diversity of *E. coli *and ii) to capture a diverse collection of a single pathogen type (EHEC O157:H7). In doing so, we were able to not only evaluate the ability of this array to measure the global genomic diversity of this species, but also to assess whether this array was useful for discriminating among individual, and closely related, strains of the same pathogen type. The latter is an important feature for the newly emerging fields of microbial forensics and molecular epidemiology, where the ability to uniquely identify and discriminate among closely related strains is of great importance in conducting attribution investigations of foodborne outbreaks or of covert biocrimes and potential bioterrorism activities [15,16].

**Table 1:** Strains Interrogated in this Study

DMB ID	Other Designation	Serotype	Pathotype	Source
EC1427	493/89	O157:H-	EHEC1	Human, Germany, 1989
EC510	4936i	O157:H-	STEC	Human
EC1231	G5101	O157:H7	EHEC1	Human, WA, 1995
EC506	ATCC43888	O157:H7	EHEC	Human
EC873	0015	O157:H7	EHEC	Human
EC1220	CAN28	O157:H7	EHEC	Human, Canada
93-111	93-111	O157:H7	EHEC1	Human, WA, 1993
95-0001A	95-0001A	O157:H7	NA	NA
EC867	0004	O157:H7	EHEC	Salami
EC4501	E2006002641	O157:H7	EHEC	Human, Taco John
EC1276	ATCC BAA-460	O157:H7	EHEC	ATCC BAA-460
EC866	0003	O157:H7	EHEC	WA
EC874	0016	O157:H7	EHEC	Apple Cider
EC877	0019	O157:H7	EHEC	Jack-in-the-box, 1993
EC868	0005	O157:H7	EHEC	NA
EC871	0012	O157:H7	EHEC	Human, AK, 1983
EC876	0018	O157:H7	EHEC	NA
EC533	86-24	O157:H7	NA	Human, WA, 1986
EC879	0023	O157:H7	EHEC	NA
EC878	0022	O157:H7	EHEC	derived from 86-24
EC883	0027	O157:H7	EHEC	NA
86-24	86-24	O157:H7	EHEC1	Human, WA, 1986
EC887	0032	O157:H7	EHEC	NA
EC881	0025	O157:H7	EHEC	mutant of 86-24
EC882	0026	O157:H7	EHEC	NA
EC535	86-01	O157:H7	EHEC	Human, WA, 1986
EC552	491	O157:H7	EHEC	Human Sizzler Steak House
EC1422	DEC3A	O157:H7	EHEC1	Human, WA, 1985
EC507	ATCC35150	O157:H7	EHEC	Human
EC1221	CAN110	O157:H7	EHEC	Human, Canada
EC1219	CAN12	O157:H7	EHEC	Human, Canada
EC1218	WETH	O157:H7	EHEC	Human, 2003
EC1222	CAN146	O157:H7	EHEC	Human, Canada
EC1217	MUS	O157:H7	EHEC	Human, 2003
EC1425	DEC3D	O157:H7	EHEC1	Human, MI, 1988
EC870	0009	O157:H7	EHEC	NA
EC516	EC269	O157:H7	EHEC	Human
EC1215	DIRKA	O157:H7	EHEC	Human, 2000
EC1226	OK-1	O157:H7	EHEC1	Human, Japan, 1996
EC512	EC262	O157:H7	EHEC	Hamburger
EC518	EC267	O157:H7	EHEC	Human
EC514	EC260	O157:H7	EHEC	PAH, CA Dept Health
EC515	EC261	O157:H7	EHEC	PAH, CA Dept Health
EC502	EC121	O157:H7	EHEC	PAH, CA Dept Health
EC1274	ATCC 43895	O157:H7	EHEC	ATCC 43895
EC1423	DEC3B	O157:H7	EHEC1	Human, WA, 1988
EC423	#260	O157:H7	NA	NA
EC503	EC177	O157:H7	EHEC	Human
EC885	0029	O157:H7	EHEC	NA
EC872	0013	O157:H7	EHEC	NA
EC504	ATCC43894	O157:H7	EHEC	Human
EC509	ATCC43890	O157:H7	EHEC	Human
EC875	0017	O157:H7	EHEC	Human
EC1214	CAI	O157:H7	EHEC	Human, 2002
EC869	0006	O157:H7	EHEC	NA
EC1212	EC536-ΔmutS	O157:H7	EHEC	EC536-ΔmutS
EC1242	48	O157:H7	EHEC	Human, GA 1992
EC536	86-17	O157:H7	EHEC	
EC513	EC263	O157:H7	EHEC	Human
EC517	EC266	O157:H7	EHEC	Human
EC508	ATCC43889	O157:H7	EHEC	Human
EC4401	06E02109	O157:H7	EHEC	Human, PA, 2006
EC1429	DEC4B	O157:H7	EHEC1	Human, Denmark, 1987
EC4001	KY 06-830	O157:H7	EHEC	Human, 2006
EC4002	KY 06-831	O157:H7	NA	Human, 2006
EC886	0031	O55:H7	EPEC	Human, WA, 1991
DEC5A	DEC5A	O55:H7	EPEC	Human, NY
ECOR37	ECOR37	ON:HN	NA	Marmoset, WA
EC1364	DEC2A	O55:H6	EPEC1	Human, Congo, 1962
EC1521	CFT073	O6:H1:K2	UPEC	ATCC 700928
ECOR56	ECOR56	O6:H1	NA	Human, Sweden
ECOR55	ECOR55	O25:H1	UPEC	Human, Sweden
EC591	ATCC35376	ON:NM	NA	Gorilla, WA
EC699	V27	O2:K5:H1	ExPEC	Human, WA
ECOR51	ECOR51	O25:HN	NA	Human, MA
ECOR23	ECOR23	O86:H43	NA	Elephant, WA
ECOR52	ECOR52	O25:H1	NA	Orangutan, WA
ECOR54	ECOR54	O25:H1	NA	Human, IA
ECOR32	ECOR32	O7:H21	NA	Giraffe, WA
EC678	H38-2906	O1:K1:H7	ExPEC	Human, WA
EC669	H15-2267	O2:K1:H7	ExPEC	Human, WA
EC674	H25-2916	O2:K1:H7	ExPEC	Human, WA
EC715	PM6	O2:K1:H7	ExPEC	Human, WA
EC728	168-2P6(B)	O2:K1:H7	ExPEC	Human, WA
ECOR61	ECOR61	O2:NM	NA	Human, Sweden
ECOR62	ECOR62	O2:NM	UPEC	Human, Sweden
ECOR59	ECOR59	O4:H40	NA	Human, MA, 1979
EC665	H5-2631	O18ac:K5:H-	ExPEC	Human, WA
ECOR64	ECOR64	O75:NM	UPEC	Human, Sweden
ECOR65	ECOR65	ON:H10	NA	Celebese ape, WA
EC1381	536	O6:H31	UPEC	Human, Model UTI, PAI
ECOR53	ECOR53	O4:HN	NA	Human, IA
ECOR60	ECOR60	O4:HN	UPEC	Human, Sweden
ECOR42	ECOR42	ON:H26	NA	Human, MA, 1979
ECOR31	ECOR31	O79:H43	NA	Leopard, WA
ECOR43	ECOR43	ON:HN	NA	Human, Sweden
ECOR35	ECOR35	O1:NM	NA	Human, IA
ECOR36	ECOR36	O79:H25	NA	Human, IA
EC716	PM7	O7:H-	ExPEC	Human, WA
ECOR40	ECOR40	O7:NM	UPEC	Human, Sweden
EC590	ATCC35360	O7:NM	NA	Human, Tonga, 1982
ECOR38	ECOR38	O7:NM	NA	Human, IA
ECOR39	ECOR39	O7:NM	NA	Human, Sweden
EC689	V14	O2:K5:H-	ExPEC	Human, WA
ECOR49	ECOR49	O2:NM	NA	Human, Sweden
ECOR50	ECOR50	O2:HN	UPEC	Human, Sweden
ECOR46	ECOR46	O1:H6	NA	Ape, WA
ECOR48	ECOR48	ON:HM	UPEC	Human, Sweden
EC1522	NBFAC05.034.01	O157	NA	Thailand, 1986
ECOR44	ECOR44	ON:HN	NA	Cougar, WA
ECOR47	ECOR47	OM:H18	NA	Sheep, New Guinea
SH20011	SH20011	dysenteriae	dysenteriae	W. Reed
SH20008	ATCC 9207	boydii	boydii	W. Reed
SH20009	53G	sonnei	sonnei	W. Reed
SH20010	2457T	flexneri	flexneri	W. Reed
EC1517	E110019	O111:H9	EPEC	Human, Finland
EC1410	MT#80	O103:H2	NA	Human; MT
EC1375	DEC12F	O111:NM	EPEC2	Human, WA, 1983
EC1370	DEC8B	O111:H8	EHEC2	Human, ID, 1986
EC1400	3007-85	O111:NM	EHEC2	Human, NE, 1985
EC1449	DEC8A	O111a:NM	EHEC2	Human, MD, 1977
EC1460	DEC10B	O26:H11	EHEC2	Human, Australia, 1986
EC400	NA	O26:H11	EHEC	Human
EC1495	H19	O26:11	EHEC2	Human
EC1497	VP30	O26:H-	EHEC2	Human, Chile, 1989
EC1496	TB285C	O26:H-	EHEC2	Human, WA, 1991
EC1395	TB285A	O26:H2	EHEC2	Human, WA, 1991
EC1459	H30	O26:H11	EHEC2	Human, UK
EC1464	RDEC-1	O15:NM	EHEC2	Rabbit, SC, 1970
EC1454	DEC9A	O26:H11	EHEC2	Human, WI, 1961
EC1457	DEC9D	O26:H11	EHEC2	Human, Denmark, 1967
ECOR66	ECOR66	O4:H40	NA	Celebese ape, WA
ECOR63	ECOR63	ON:NM	NA	Human, Sweden
EC592	ATCC35386	O4:H43	NA	Goat, Indonesia
ECOR24	ECOR24	O15:NM	NA	Human, Sweden
ECOR70	ECOR70	O78:NM	NA	Gorilla, WA
ECOR72	ECOR72	O144:H8	UPEC	Human, Sweden
EC718	PM9	O9:K34:H-	ExPEC	Human, WA
ECOR71	ECOR71	O78:NM	NA	Human, Sweden
ECOR58	ECOR58	O112:H8	NA	Lion, WA
ECOR69	ECOR69	ON:NM	NA	Celebese ape, WA
ECOR68	ECOR68	ON:NM	NA	Giraffe, WA
EC319	B7A	O148:H28	ETEC	NA
ECOR7	ECOR7	O85:HN	NA	Orangutan, WA
EC1523	NBFAC05.034.02	O157	NA	Thailand, 1986
ECOR34	ECOR34	O88:NM	NA	Dog, MA
ECOR29	ECOR29	O150:H21	NA	Kangaroo rat, NV
ECOR33	ECOR33	O7:H21	NA	Sheep, CA
EC589	ATCC35349	O113:H21	NA	Bison, Canada
ECOR26	ECOR26	O104:H21	NA	Human, MA
ECOR27	ECOR27	O104:NM	NA	Giraffe, WA
ECOR28	ECOR28	O104:NM	NA	Human, IA
ECOR45	ECOR45	ON:HM	NA	Pig, Indonesia
EC1490	MG1655	OR:H48:K-	NA	ATCC 47076
MG1655-mutS	MG1655-ΔmutS	OR:H48:K-	NA	MG1655-ΔmutS
EC1216	FULLE	NA	NA	Human, 2003
ECOR6	ECOR6	ON:HM	NA	Human, IA
ECOR25	ECOR25	ON:HN	NA	Dog, NY
ECOR10	ECOR10	O6:H10	NA	Human, Sweden
ECOR8	ECOR8	O86:NM	NA	Human, IA
ECOR1	ECOR1	ON:HN	NA	Human, IA
ECOR3	ECOR3	O1:NM	NA	Dog, MA
ECOR18	ECOR18	O5:NM	NA	Celebese ape, WA
EC1223	CAN9139	NA	NA	Human, Canada
ECOR14	ECOR14	OM:HN	UPEC	Human, Sweden
ECOR9	ECOR9	ON:NM	NA	Human, Sweden
ECOR12	ECOR12	O7:H32	NA	Human, Sweden
ECOR5	ECOR5	O79:NM	NA	Human, IA
ECOR11	ECOR11	O6:H10	UPEC	Human, Sweden
ECOR2	ECOR2	ON:H32	NA	Human, NY, 1979
ECOR13	ECOR13	ON:HN	NA	Human, Sweden
ECOR20	ECOR20	O89:HN	NA	Steer, Bali
ECOR21	ECOR21	O121:HN	NA	Steer, Bali
EC563	ATCC43886	O25:K98:NM	ETEC	Human
ECOR19	ECOR19	O5:NM	NA	Celebese ape, WA
EC164	4608-58	O143	EIEC	NA
EC568	ATCC43893	O124:NM	EIEC	ATCC 43893
EC884	0028	O55:H7	EPEC	Human, WA, 1991
ECOR15	ECOR15	O25:NM	NA	Human, Sweden
ECOR16	ECOR16	ON:H10	NA	Leopard, WA
ECOR22	ECOR22	ON:HN	NA	Steer, Bali
ECOR17	ECOR17	O106:NM	NA	Pig, Indonesia
ECOR4	ECOR4	ON:HN	NA	Human, IA

When a strain history is unknown, we used a "NA" designation.

The probe set design utilized in this expression array allowed highly accurate determination of a gene allele presence. These data, combined with a unique custom analysis approach, eliminate the need of a reference strain, which previously has been an absolute requirement for accurate comparative genomic hybridization (CGH) studies. The comprehensive wealth of data derived from this study has been used to identify groups of genes that define a particular pathogen type. The use of this microarray approach, combined with the large sampling size, gives insight to global *E. coli *diversity on a previously unexplored scale.

## Methods

### Bacterial strains and preparation of genomic DNA

Strains of *E. coli *and *Shigella *used in this study are listed in Table 1. Strains EDL933, MG1655, and CFT073 are *E. coli *O157:H7, K-12, and uropathogenic strains, respectively, for which the genome sequences are available [17-19]. Sakai is a Japanese enterohemorrhagic O157:H7 outbreak strain, and likewise the genome sequence is available [20].

Strains were grown overnight in 3 mls of Luria Broth at 37°C in a shaking incubator. Genomic DNA was isolated from 2 mls of the overnight culture using the Qiagen DNeasy Tissue kit following the manufacturer's recommendations. Typically, 5-10 μg of purified genomic DNA was recovered in a final elution volume of 200 μl. The purified DNA was further concentrated using Microcon YM-30 microcentrifuge filters to a final volume of approximately 10 μl. 5 μg of the genomic DNA was fragmented by incubating at 37°C for 10 minutes in a 50 μl reaction containing 1X One-Phor-All Plus Buffer (GE Healthcare) and 0.1 units DNase I (GE Healthcare). The fragmentation reaction was heat-inactivated at 95°C for 10 minutes. Following fragmentation, the DNA was 3'-end labeled by adding 4 μl of 5X terminal transferase buffer (Promega), 1 μl of 1 mM biotin-11-ddATP (PerkinElmer NEL508), and 2 μl (60 units) of terminal transferase enzyme (Promega) (final volume 27 μl). Labeling was carried out for at least 2 hours at 37°C followed by heat inactivation at 95°C for 10 minutes.

### Array hybridization, washing, staining, and scanning

Hybridizations were performed according to the Affymetrix GeneChip Expression Analysis Technical Manual for the 169 format array [21]. Briefly, 80 μl hybridizations containing 5 μg of fragmented/labeled DNA, 100 mM MES, 1 M [Na+], 20 mM EDTA, 0.01% Tween-20, 50 pM control oligo B2, 0.1 mg/ml salmon sperm DNA (Sigma), 7.8% DMSO (Sigma), and 0.5 mg/ml BSA (Sigma) were hybridized onto the Affymetrix GeneChip^® ^*E. coli *Genome 2.0 Array, incubated at 45°C, with rotation (60 rpm) for 16 hours in a hybridization oven.

Following hybridization, the wash and stain procedure was carried out on an Affymetrix FS-450 fluidics station using the mini_prok2v1_450 fluidics script [21]. Wash and stain reagents were prepared according to the GeneChip^® ^Expression Analysis Technical Manual [21]. The following exceptions were made to the wash and stain procedure: Streptavidin solution mix (vial 1) was replaced with SAPE solution mix. Arrays were scanned using a GeneChip^® ^Scanner 3000 7G running GCOS v1.4 software.

### Analysis Considerations for Genotyping with the Affymetrix *E. coli *2.0 Array

Adapting an expression array for genotyping purposes required a novel data analysis approach, given that the use of the Affymetrix probe set design produces 22 independent measurements for each genome target. These 22 measurements are derived from a single probe set, consisting of 11 probe pairs. Each probe pair is composed of a single 25-mer perfect match (PM) oligo and its corresponding 25-mer mismatch (MM) oligo. The mismatch oligo is identical to the perfect match with the exception of a single nucleotide mismatch located at the central (13^th^) position of the oligo sequence. Therefore summarizing these independent measurements into a single probe set intensity value that is indicative of the status of a particular gene can be problematic. This can be further complicated when one or more of the individual probes (25-mers) in a probe set is able to hybridize to another genomic region, thereby giving rise to a "partially specific" probe set. Secondly, the Affymetrix array is a multi-genome array. Previous CGH studies have required a reference strain in order to accurately detect whether a particular gene target was present/absent. For a multigenome array, however, it is not possible to use data from any single hybridization experiment as a reference data set. Therefore the status of each gene target should, ideally, be determined by considering only raw probe intensities from an individual experiment.

### Parsing CEL Files and Data Analysis Tools

All 207 Affymetrix CEL files generated in this study were parsed using the Robust MultiArray Averaging (RMA) method (Bioconductor affy Package and Affymetrix Power Tools) [22-25]. Hierarchical clustering analysis and principal component analysis was done using Spotfire and the MADE4 package of Bioconductor [26,27]. MAS 5.0 gene present/absent calls were determined using Affymetrix Power Tools as well as the affy Bioconductor Package [24,28]. All data files, including 207 Affymetrix CEL files, the RMA-summarized probe set intensities, and the MAS 5.0 gene present/absent calls, can be accessed at http://www.mrscentral.com

### Probe Set Summarization Methods

It is desirable to determine the summarized intensity of each probe set (consists of 11 PM and 11 MM oligos). In all of our analyses, summarized probe set intensities were calculated for each strain by using the Robust MultiArray Averaging (RMA) [23] method as implemented in the affy package of Bioconductor or Affy Power Tools. In brief, RMA summarization of probe level data is done by performing three individual treatments on all of the experimental CEL data simultaneously. First, probe specific correction of the PM probes is done using a model based on the observed intensities being the sum of signal and noise. Secondly, quantile normalization is performed on the corrected PM probe intensities. Finally, a median polishing algorithm is used to summarize the background-corrected, normalized probe intensities to generate a final probe set value.

We also evaluated the utility of the MAS 5.0 probe set summarization algorithm [28]. Briefly, the signal is calculated as follows: i) global background correction, ii) ideal MM value is calculated and subtracted to adjust the PM intensity, iii) adjusted PM intensities are log-transformed to stabilize the variance, iv) biweight estimation to provide a robust mean of the resulting values, and finally v) probe set intensity signal is scaled using a trimmed mean.

### Making Accurate Gene Present/Absent Calls

In addition to summarizing probe set intensities, it is also desirable to make absolute gene present/absent calls. We assessed two novel methods for determining the status of each gene using the hybridization intensities from the four *E. coli *strains that are represented on this array. The first method was included in the standard Affymetrix GCOS analysis package and is referred to as the MAS 5.0 Gene Detection approach [28]. Here gene targets are determined to be either present, absent, or marginal as determined by a p-value calculated from the discrimination score (R) for each probe pair. The discrimination score is a basic property of a probe pair that describes its ability to detect its intended target. It measures the target-specific intensity difference of the probe pair (PM-MM) relative to its overall hybridization intensity (PM+MM). The discrimination score (R) is therefore defined as:  and approaches 1.0 as the mismatch probe intensity approaches 0.

The next step in calculating a detection p-value is to compare each discrimination score to the user-definable threshold Tau. Tau is a small positive number that can be adjusted to increase or decrease sensitivity and/or specificity of the analysis.

Increasing the threshold Tau can reduce the number of false positive (present) calls but may also reduce the number of true present calls. We found that the most accurate gene detection calls were made when using a Tau value of 0.20. Next, the one-sided Wilcoxon Signed Rank test is employed to generate the detection p-value. It assigns each probe pair a rank based on how far the probe pair discrimination score is from Tau. The detection p-value is then compared to a user-definable cutoff value that results in the final present/absent call for each gene. Here again, we evaluated several cut-off values for the detection p-value and found that a value of 0.050 provided highly accurate gene detection calls. That is, probe sets with detection p-values <0.05 were scored as "present", and greater than, or equal to, 0.05 as "absent".

The second method that we used for determining the status of a particular gene target is analogous to the more conventional methods of CGH where a reference strain is used to determine relative hybridization intensities. Here, we calculated a "RefMax" value for each probe set by determining the maximum hybridization intensity (RMA summarized) from each of the four reference strains represented on the array. Therefore, "RefMax" represents the maximum observed hybridization intensity for each probe set as determined by the appropriate reference strain to which the probe set was designed (for example, the RefMax for probe set j is calculated as: RefMax_j _= Max(CFT073_j_, EDL933_j_, Sakai_j_, MG1655_j_)). Absolute probe set intensities for each of the 207 hybridization experiments performed were compared to the RefMax data. For strain i, probe set j, RefMax Ratio is defined as:

For each probe set, gene targets were scored as "absent" if their hybridization intensities were greater than 4-fold lower from the RefMax value. Otherwise, genes were scored as "present".

### Phylogenetic Analysis of *E. coli *Genome Data

Tables containing 10,208 MAS 5.0 Present/Absent calls (described above) were transformed into A/T binary nucleotide calls for each isolate. Tables were then wrapped/concatenated into fasta-formatted text files. Fasta text files were then used directly as input for the MEGA5 software package [29]. Phylogenetic analysis was performed using the maximum likelihood method.

## Results

In the present study, we interrogated the genomic content of 207 isolates of *E. coli *and *Shigella *using the Affymetrix GeneChip^® ^*E. coli *Genome 2.0 Array, choosing to explore both the level of diversity that existed among closely related strains of the same pathotypes (i.e. independent O157:H7 strains) and the level of genomic diversity that existed among a globally diverse collection of *E. coli *strains (i.e. the ECOR collection). From this analysis, we were able to determine, with high accuracy, the gene content of each of these strains relative to those probes represented on the array.

### Probe Set Summarization Methods: RMA *vs*. MAS 5.0

**Figure 1:**
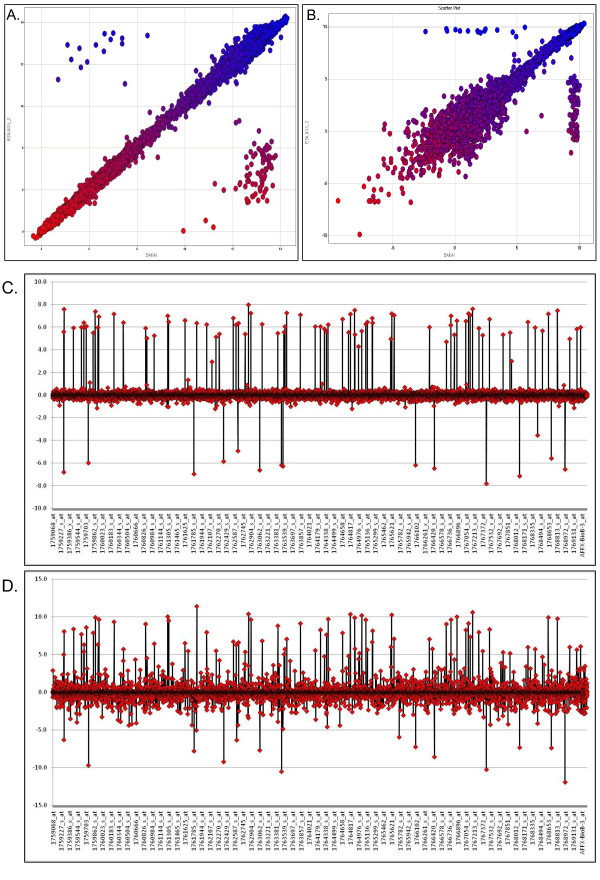
**Comparing microarray probe set summarization methods: RMA *vs*. MAS 5.0 from two sequenced references strains of *Escherichia coli *O157:H7 (EDL933 and Sakai)**. (A.) Scatter plots showing RMA summarized probe set intensities from strains EDL933 (y-axis) and Sakai (x-axis). (B.) Scatter plots showing MAS 5.0 summarized probe set intensities from strains EDL933 (y-axis) and Sakai (x-axis). In both A. and B., data points are color-coded based on their intensities in EDL933. (C.) Line plot showing EDL933 RMA intensity relative to Sakai RMA intensity (log_2_[EDL933]/[Sakai]). (D.) Line plot showing EDL933 MAS 5.0 intensity relative to Sakai MAS 5.0 intensity (log_2_[EDL933]/[Sakai]).

In Figure 1, we demonstrate the advantage of using RMA over MAS 5.0 by comparing summarized probe set intensities from the two reference O157:H7 strains, EDL933 and Sakai. From the scatter plots in Figure 1, it is apparent that the RMA probe summarization method (Figure 1A) yields a much lower variance in probe sets where intensities are less than 8 (log2) as compared to MAS 5.0 probe set summarization method (Figure 1B). This decrease in variance allows for a more accurate determination of actual gene differences between these two strains. This is even more apparent when relative probe intensities (log_2_[Sakai]/[EDL933]) are plotted. Summarized probe set intensities from the MAS 5.0 method result in large fold-change differences (Figure 1D), suggesting, incorrectly, the presence of genomic differences at these loci (false positives). However, RMA's ability to reduce this variability (Figure 1C) provides a much higher confidence in accurately identifying true genomic differences (fewer false positives). Because we feel that RMA is a better method for summarizing probe set intensities, we performed all downstream analyses (hierarchical clustering, Pearson correlation matrix, and PCA) using RMA summarized probe set intensity data.

### Determining the Status of Gene Targets: RefMax *vs*. MAS 5.0

Using RefMax we found that >99.85% (5653/5654) of the target genes that shared >98% sequence homology to a particular probe set were accurately detected as "present" as shown in Table 2. Also, 64% of the genes sharing 96%-98% sequence homology are called "present" and when homology decreases to between 94%-96%, only 27% of the probe targets are called "present". Here we are not always attempting to score these present/absent calls as either "correct" or "incorrect" but rather simply report our finding that the probe set design used here is capable of discriminating among closely related genes sharing >90% sequence homology [30]. Also shown in Table 2, using MAS 5.0, 99.83% of the probe sets having >98% homology to their target gene were scored correctly as "present". Further, 55% of the probe sets having between 96%-98% sequence homology to their gene target were called as "present". In comparison to the MAS 5.0 method, the RefMax method is more sensitive to sequence variation between the probe set and its gene target. This clearly can be seen when sequence homologies between probe sets and gene targets are relatively low (between 90%-92%). In these instances, the MAS 5.0 method called 22.4% of the gene targets as "present" whereas our RefMax method only called 5.6% of these gene targets as "present". Furthermore, a clear advantage of using the MAS 5.0 method for scoring genes was its ability to accurately call genes as being "present" regardless of the overall probe intensities. For example, in three instances, where gene target lengths were below 100 nt and corresponding probe set intensities were in the bottom 2% of all probe set intensities on the array, the Affymetrix MAS 5.0 method correctly predicted the status of these gene targets in each of the reference strains a total of 19 out of 21 times. This corresponds to an accuracy of 90% (10% false negatives) for probe sets having intensities in the bottom 2% of all probe sets.

**Table 2:** RefMax vs. MAS 5.0: A Validation Study

			Present		Absent	
Genome	Homology Bin	Genes Present	MAS5	RefMax	MAS5	RefMax
NC_000913.2	100%	5654	5651/5651	5653/5654	3/3	1/0
NC_000913.2	>98%	391	385/385	382/382	6/6	9/9
NC_000913.2	>96%	410	263/268	225/226	147/142	185/184
NC_000913.2	>94%	347	92/102	49/48	255/245	298/299
NC_000913.2	>92%	202	36/35	13/13	166/167	189/189
NC_000913.2	>90%	143	32/36	8/9	111/107	135/134
NC_000913.2	<90%	12	3/3	1/1	9/9	11/11
						
NC_002655.2	100%	3569	3565/3564	3558/3566	4/5	11/3
NC_002655.2	>98%	2656	2653/2653	2644/2648	3/3	12/8
NC_002655.2	>96%	1178	1053/1054	963/963	125/124	215/215
NC_002655.2	>94%	502	302/287	159/162	200/215	343/340
NC_002655.2	>92%	288	143/138	59/60	145/150	229/228
NC_002655.2	>90%	231	132/132	70/69	99/99	161/162
NC_002655.2	<90%	20	8/9	6/6	12/11	14/14
						
NC_002695.1	100%	3473	3471/3472	3466/3464	2/1	7/9
NC_002695.1	>98%	2655	2652/2652	2646/2641	3/3	9/14
NC_002695.1	>96%	1164	1036/1031	945/936	128/133	219/228
NC_002695.1	>94%	511	302/291	169/167	209/220	342/344
NC_002695.1	>92%	291	140/138	66/66	151/153	225/225
NC_002695.1	>90%	208	108/102	52/52	100/106	156/156
NC_002695.1	<90%	18	7/6	5/5	11/12	13/13
						
NC_004431.1	100%	3112	3111/-	3111/-	1/-	1/-
NC_004431.1	>98%	1816	1816/-	1814/-	-/-	2/-
NC_004431.1	>96%	1585	1494/-	1450/-	91/-	135/-
NC_004431.1	>94%	509	304/-	247/-	205/-	262/-
NC_004431.1	>92%	261	96/-	55/-	165/-	206/-
NC_004431.1	>90%	198	90/-	48/-	108/-	150/-
NC_004431.1	<90%	16	7/-	6/-	9/-	10/-

Gene present/absent calls were determined for the 4 sequenced reference strains represented on the array using either the RefMax or MAS 5.0 gene detection methods. Genome corresponds to the accession number of the genome/strain being interrogated. Homology Bin corresponds to the percentage by which a probe set consensus sequence matches the target genome sequence. Genes Present corresponds to the number of genes present on the array which fall into a particular Homology Bin (this is also the maximum number of correct "present" calls). Present or Absent calls were determined from either the MAS 5.0 or RefMax method and are shown under the "MAS5" and "RefMax" headings. Strains MG1655, EDL933, and Sakai were each performed in duplicate to show the reproducibility of each method. Independent measurements are indicated by a "/" under the Present and Absent headers.

While having the advantage of being able to detect genes correctly despite sometimes having low absolute probe intensities, the MAS 5.0 method was found to be only 95% reproducible when comparing replicate experiments of a single reference strain. When "absent" and "marginal" calls were not considered separately, however, this reproducibility increased to 97% (i.e. "A" or "M" = "A/M") (as in Table 2). When examining the reproducibility of our RefMax method, we observed that well over 99.9% of all gene status calls from replicate experiments agreed. This level of reproducibility was based on whether at least a 4-fold difference was observed for any probe set from replicate experiments. The lack of reproducibility in the MAS 5.0 method appears to be a function of the level of gene homology and not necessarily absolute probe signal, whereas our RefMax method was independent of either of these factors.

When using our RefMax method for making gene status calls, it is necessary to apply a user-defined cut-off value for RefMax in order to account for probe sets that are present on the *E. coli *v2.0 array, yet absent in all four of the reference strains represented on this array. We have observed a total of 145 probe sets on this *E. coli *2.0 array which do not appear to have target genes present in any of the four reference strains. This was confirmed by BLASTing these probe set sequences against the reference genome sequences. As expected, the RefMax intensity was found to be in the lower 1.5% of all probe intensities which suggests an "absent" gene target. Of these 145 probe sets, 72 corresponded to Affymetrix control probes (probe sets designated with "AFFX-" prefix). The remaining 73 probe sets corresponded to genes encoded by various phages, transposons, plasmids as well as several known antibiotic resistance genes. These probe sets were included by Affymetrix due to their relevance to microbial pathogens. Therefore, RefMax values whose absolute probe set intensities were < 9 were considered to be absent in the reference strains. We therefore scored all probe sets from non-reference strains that differed by less than 4-fold from RefMax as "absent". Only when a non-reference strain was found to have an absolute probe set intensity 4-fold greater than the RefMax value were these genes scored as "present".

Likewise, one can use the MAS 5.0 technique described above to make present/absent calls. Upon doing so, we found that among the four reference strains, there were a total of 151 probe sets that were consistently called absent. Of these 151 probe sets, 73 corresponded to Affymetrix control probe sequences, leaving 78 other (non-control) probe sets. One can then expand this analysis from the four reference strains to all 207 isolates analyzed in this study. Upon doing so, we found 44 (non-control) probe sets that were consistently absent in all 207 *E. coli *and *Shigella *isolates examined in this study. Again, these genes were mainly antibiotic resistance elements from species other than *E. coli *or *Shigella*. Two notable exceptions were nine independent probe sets targeting Phage M13 genes as well as the EDL933 gene Z2261 (and unknown protein associated with *Rhs *element).

As an additional confirmatory method, we compared our MAS 5.0 gene present/absent calls with those determined by confirmatory PCR. The *stx1 *and *stx2 *genes were chosen as targets. We interrogated 195 isolates for the presence of these two genes by confirmatory PCR. We found an agreement between present/absent calls in 193/195 cases (99%). The two discrepancies are thought to be due to primer/probe specificity towards different *stx *alleles (data not shown).

## Discussion

### Strain Attribution, Identification, and Discrimination within the O157:H7 Pathotype

The genomic content of 63 unique strains (75 isolates) of *E. coli *O157:H7 was assessed in order to determine whether we could accurately distinguish between independent isolates of the same serotype. From this study, we found that, on average, individual strains of O157:H7 differed by 197 gene targets (Additional File 1). Moreover, among this same group of isolates, we found that a maximum of approximately 750 gene targets differed among any two independent O157:H7 isolates (EC1214 *vs*. EC885). When individual strains were examined in replicate, we found that a maximum of three gene target differences may occur between replicates. Note, however, that these "replicates" could often be independent isolates of the same strain and the observed gene differences may sometimes be due to known deletions that are present in strains that were derived from a parent strain (*i.e*. EC536 and EC1212 are isogenic). Next we tested if any of our independent isolates were indistinguishable using this array-based method, *i.e*., were there multiple isolates of the same strain. We found several instances where there were fewer than 10 gene target differences between two independent isolates (*e.g*. EC1423 *vs*. EC423, EC514 *vs*. EC515 from Additional File 1). In fact, we were able to make correlations between isolates that were previously unknown and presumed to be independent. For example, strain 86-24 (a human isolate from Washington State in 1986) differed from strain EC533 by a single gene difference. Upon further inspection of the history of these strain designations, we found that EC533 was also a human isolate from Washington State, also obtained in 1986. It is therefore likely that these two isolate designations actually refer to the same strain that were received by our laboratory from two different repositories. This precipitated a closer examination of the history of our strain collection, which showed a few similar examples of this. From a forensic perspective, this is a very important finding as it suggests that this array-based method is not only a powerful tool for discriminating among different strains of the same serovar but also provides, with a high level of confidence, a robust means for strain identification and attribution.

### Exploring the Global Genomic Diversity of *E. coli*

The ECOR collection is a set of 72 reference strains of *E. coli *isolated from a variety of hosts and geographical locations [12]. It was established for use in studies of variation and genetic structure in natural populations and is representative of the range of phenotypic variation in the species as a whole. We therefore used this well-studied collection to analyze the performance of our array-based method in measuring the global diversity of *E. coli*. Among this collection, we found that individual strains differed, on average, by approximately 1900 gene target differences. Moreover, a maximum of 4002 gene target differences was observed between any two strains (ECOR57 *vs*. ECOR37). This represents approximately 40% of the probe targets present on this array. In addition to the ECOR reference collection and our O157:H7 reference collection, we included 50 other *E. coli *and *Shigella *isolates in our analysis (Table 1). It is interesting to note that some of the most diverse strains included in this study were among the latter non-ECOR, non-O157 strains. For example, the greatest number of probe set differences, 4890, was observed between an O157:H7 strain EC885 and the UPEC CFT073 strain EC1521. While this number may seem impossibly high, it is in fact representative of gene differences and allelic differences that exist between these two evolutionarily distinct lineages of *E. coli*. Different alleles are often represented by different probe sets on the *E. coli *Genome 2.0 Array. Therefore, probe set differences do not always directly correspond to true gene differences. A gene differences matrix was prepared by calculating the number of gene differences that exists among all 207 isolates examined in this study (Additional File 1). Similarly, we performed a Pearson correlation analysis on all of the strains included in this study based on their RMA probe set summarized values. Results from this analysis depict a strain-to-strain relatedness quantified from 0 to 1 (1 being identical). A visual representation of this correlation matrix is shown in Additional File 2 and is color-coded based on relatedness.

In order to better understand the global diversity of *E. coli*, we performed several cluster-based analyses. Using the RMA-summarized data generated from all of the probe sets, we performed a hierarchical clustering analysis (Euclidean means) on the strains and gene targets to demonstrate graphically the level of genomic diversity that exists within this species (Additional File 3). The dendrogram in Additional File 3 shows that the 207 isolates examined in this study cluster into three major groups. The first cluster (left most) represents EHEC1 strains. The other two clusters cannot be clearly defined by a particular set of traits and contain an assortment of both serotypes and pathotypes.

**Figure 2:**
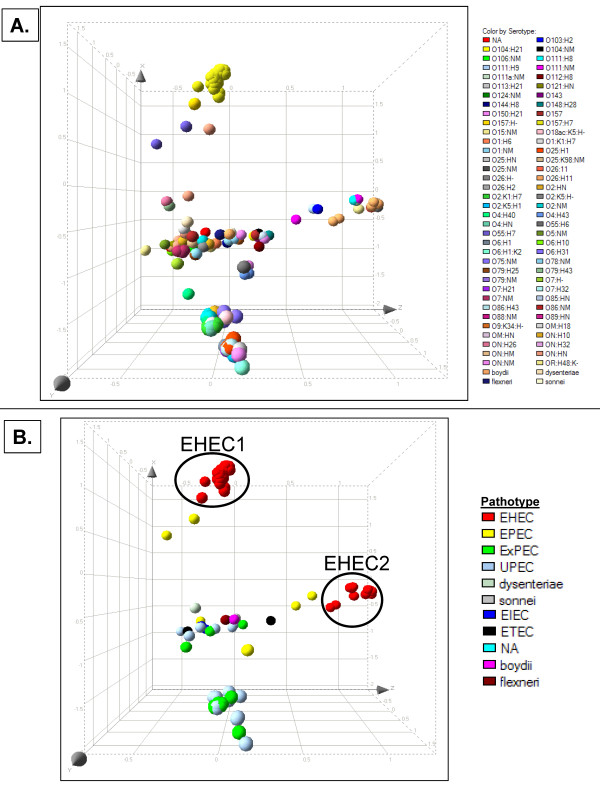
**Principle Component Analysis (PCA): The MADE4 package of R-Bioconductor was used to perform PCA on RMA-summarized probe set intensities**. The first 3 components were plotted using Spotfire. **A**. All 207 isolates are shown and color-coded based on their serotype. **B**. 128 isolates are plotted and color-coded based on their known pathotype.

To further demonstrate the relatedness and diversity of the strains examined in this study, we performed a Principal Component Analysis (PCA) on RMA-summarized probe set intensities from all 207 isolates. Upon plotting the first three dimensions of the PCA data, we color-coded the strain identifiers based on their serotype (Figure 2A). From this, we observed that strains belonging to the same serotype often, but not always, cluster together. It is worth noting that all serotypes are not equally represented in the collection of isolates that were examined in the current study, therefore it is not possible to make conclusions regarding the clustering of some serotypes. As pathotype information was available for 128 of our isolates, a PCA was performed on these isolates independently (Figure 2B). This PCA plot showed strong clustering of isolates from the EHEC1 and EHEC2 pathotypes. Though these pathotypes have similar clinical manifestations, their genomes are quite distinct. While some strain of the same pathotype often co-clustered, this was not universally true, therefore suggesting that virulence traits are inherited horizontally. Indeed, this phenomenon has been reported previously [31,32].

### Examining the Conserved, Core, Backbone Genes of *E. coli*

Our array-based technique is well suited for determining which genes are conserved in various types of *E. coli*. Initially, we wanted to determine which gene targets were present in all strains of *E. coli *and *Shigella *included in this study. We found 2256 gene targets, using the MAS 5.0 technique described above, that were consistently present in all 207 isolates examined (Additional File 4). We define these gene targets as being the core/conserved genic regions that make up the *E. coli *genomic backbone. Similar analyses have been performed previously on whole genome sequence data [33]. From these *in silico *analyses, approximately 2200 conserved core genes were identified. Therefore, previous *in silico *analyses correlate well with our microarray-based approach.

Moreover, because the Affymetrix GeneChip^® ^*E. coli *Genome 2.0 Array represents intergenic regions from the MG1655 genome, we were also able to assess the conserved intergenic regions as well. In total, there are 686 unique probe sets representing 240.9 kb of intergenic sequences (Additional File 5). Of these, we found 232 unique probe sets that were conserved (always present) in all 207 isolates analyzed. These 232 probe sets represent 76.8 kb of conserved intergenic sequences. Assuming an average genome size of 5 Mb, this suggests that strains of *E. coli *share approximately 1.5% of conserved intergenic sequences.

### Defining the EHEC Pathotype-Specific Genes

Because we examined a large number (63) of O157:H7 strains and a large number of non-O157:H7 strains, we are also able to make conclusions on which genes are responsible for defining the O157:H7 serotype. That is, which genes are always present in O157:H7 strains but never present in non-O157:H7 strains. For this analysis, we included only O157:H7 strains and excluded O157:H- and "O157" (EC1522, EC1523, EC510, EC1427) strains. As might be expected, most genes fitting this criterion engender information for the biosynthesis of the O and H antigens (Table 3).

**Table 3:** O157:H7-Specific Gene Targets

ProbeSet ID	Accession ID	Strain	Genome Position	Locus	Gene	Product	GI
1766456_s_at	NC_002655.2	Escherichia coli O157:H7 EDL933	2849958-2851076	Z3198-RC	-	EDL933	16445223
1759686_s_at	NC_002655.2	Escherichia coli O157:H7 EDL933	2848990-2849955	Z3197	fcI	fucose synthetase	962092
1759686_s_at	NC_002695.1	Escherichia coli O157:H7 Sakai	2778776-2779741	ECs2838	-	fucose synthetase	912293
1766456_s_at	NC_002695.1	Escherichia coli O157:H7 Sakai	2779744-2780862	ECs2839	-	GDP-D-mannose dehydratase	912548
1766456_s_at	NC_002655.2	Escherichia coli O157:H7 EDL933	2849958-2851076	Z3198	-	GDP-mannose dehydratase	962093
1764806_s_at	NC_002655.2	Escherichia coli O157:H7 EDL933	2848478-2848987	Z3196	wbdQ	GDP-mannose mannosylhydrolase	962091
1762793_s_at	NC_002695.1	Escherichia coli O157:H7 Sakai	2780369-2780601	ECs5479	-	hypothetical protein	2693774
1759443_s_at	NC_002695.1	Escherichia coli O157:H7 Sakai	2776834-2778282	ECs2836	-	mannose-1-P guanosyltransferase	912820
1759443_s_at	NC_002655.2	Escherichia coli O157:H7 EDL933	2847048-2848496	Z3195	manC	mannose-1-P guanosyltransferase	962090
1762953_s_at	NC_002695.1	Escherichia coli O157:H7 Sakai	2783218-2784603	ECs2842	-	O antigen flippase	912601
1762953_s_at	NC_002655.2	Escherichia coli O157:H7 EDL933	2853432-2854823	Z3201	wzx	O antigen flippase Wzx	962096
1766849_s_at	NC_002655.2	Escherichia coli O157:H7 EDL933	2855525-2856709	Z3203	wzy	O antigen polymerase	962098
1766849_s_at	NC_002695.1	Escherichia coli O157:H7 Sakai	2785311-2786495	ECs2844	-	O antigen polymerase	912486
1764806_s_at	NC_002695.1	Escherichia coli O157:H7 Sakai	2778264-2778773	ECs2837	-	putative GDP-L-fucose pathway enzyme	912421

Using the MAS 5.0 gene detection method, we filtered those probe sets that were consistently called "present" in all O157:H7 strains yet were called "absent" in all non-O157:H7 strains. The 14 probe sets shown here correspond to O157:H7-specific genes.

### Phylogenetic Analysis of E. coli Genomes Using Microarray Data

**Figure 3:**
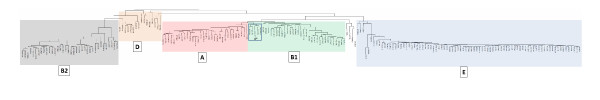
**Molecular Phylogenetic analysis by Maximum Likelihood method: The evolutionary history was inferred by using the Maximum Likelihood method based on the Tamura-Nei model **[35]. The tree with the highest log likelihood (-282250.6332) is shown. Initial tree(s) for the heuristic search were obtained automatically as follows. When the number of common sites was < 100 or less than one fourth of the total number of sites, the maximum parsimony method was used; otherwise BIONJ method with MCL distance matrix was used. The tree is drawn to scale, with branch lengths measured in the number of substitutions per site. There were a total of 10208 positions in the final dataset.

Extant populations of *E. coli *are structured into main phylogenetic groupings. The evolutionary history of *E. coli *has usually been recapitulated employing Multi Locus Sequence Typing (MLST) analysis of the ECOR collection. However, it has been shown that a correlation exists between the phylogenetic history of the strains and the presence/absence of genes. We compared our whole-genome microarray analysis approach to the main phylogenetic groups of the ECOR collection previously defined and based on MLST data. For the most part, our whole genome analysis of the ECOR collection correlated well with previous designated phylogenetic groups as shown in Figure 3. There were, however, several notable exceptions. For example, strains ECOR70, ECOR71, and ECOR72 that were previously defined in group B1 were, by our analysis, found to be part of group A (shown in Figure 3 as A*). And, notably, as with MLST, array analysis showed group D strains to be more heterogeneous than other groupings, seemingly reflecting diverse and distinct lineages of Group D strains.

One might *a priori *expect disagreement between our whole-genome approach and previous MLST analyses. That is, whereas MLST is an approximation of the mutation rate of the genomic backbone based on sequence analysis of a relatively small number of housekeeping genes, analysis of whole genome differences is a measure of both the rate of mutation and horizontal gene transfer that exists among this species. These two analyses are quite different and distinct. So it is therefore interesting to find such a remarkably high level of correlation between these two different approaches.

## Conclusions

The current study is an in depth analysis of the genomic content of a large and diverse collection of *E. coli *strains, including strains of the closely related genus *Shigella*. By utilizing a high-density oligo array representing 10,112 unique probe sets, we were able to qualitatively describe the complete gene repertoire of each of 183 unique strains. In so doing, we found that diverse strains can differ by as many as 4890 probe set targets, *e.g*., CFT073 strain EC1521 and the O157:H7 strain EC885. We found that, among the 207 independent isolates examined in the present studies, individual strains differed, on average, by 2276 probe sets, and the median number of probe set differences was 2597. When one focuses on only the EHEC-like isolates (Additional File 3, cluster 1), we found that there are 2321 probe sets that are variable among this EHEC cluster. On average, EHEC cluster 1 strains differed by 197 probe sets.

We also examined how our microarray-based method was able to recapitulate the phylogenetic history of isolates. Using the maximum-likelihood method, we found a remarkable level of correlation between previous MLST-based results and our current microarray-based results. While these two approaches differ, in some cases however, these two analyses may be regarded as highly similar. Many of the probe sets on the *E. coli *Genome 2.0 array are in fact representatives of different alleles of the same gene. We give as an example the *mutS *gene: the *mutS *gene is >95% conserved in nucleotide sequence in strains Sakai, MG1655, and CFT073. However, on the *E. coli *2.0 array, there are three different probe sets that represent these three allelic variants of *mutS*. Importantly, the hybridization-based strategy utilized here is capable of discriminating between these three alleles [30]. So, indeed, our microarray approach is, in some cases, is also a measure of allelic diversity that exist among housekeeping genes. This is highly analogous to an MLST-based assay.

It is tempting to speculate on the minimal number of gene differences observed between any two independent isolates derived from the same culture. However, we will refrain from making any specific qualitative remarks to this regard. The reason is simply one of semantics in that the definition of "independent isolates" varies based on perspective. For example, a clinician might refer to two isolates derived from the same patient on different days as being "independent". Yet from a genomics point of view, these two isolates may well be the same strain as measured by their genomic content.

The concept and definition of "independent isolates" and "different strains" came to light in our investigation of the 2006 outbreak of O157:H7 associated with fresh spinach. During this outbreak, we interrogated >200 "independent isolates" that were geographically diverse (different clinical isolates from different states across the country or various food isolates). Collectively, we referred to these isolates as the "outbreak population". All of the isolates contained within this outbreak population, with the exception of two, were genomically indistinguishable (no probe set differences). The two exceptions differed by a single phage-related insertion. This difference resulted in the appearance of approximately 40 probe set differences, a result later confirmed independently in our laboratory by optical mapping and whole genome sequencing [34]. So, the question of minimal gene differences can only be answered accurately in the context of the whole genome. Whereas these 40 gene differences might indicate a new strain, clearly they were derived from the same population of *E. coli *O157:H7 that caused the outbreak; i.e., they were indeed siblings within the outbreak population.

It is imperative to consider, as technology allows us to delve deeper into the bacterial genome, that individual strains and independent isolates are not synonymous terms. That deriving isolates from a single sick individual on different days; diluting and plating a single cultured sample onto several plates and subsequently deriving isolates from different plates; deriving isolates from different patients sickened during a single foodborne outbreak; or obtaining isolates from different environmental samples, are all valid examples of what "independent isolates" denote. These independent isolates, however, may, but need not, be individual strains. That is, if differences, no matter how subtle, are observed in genome comparisons of two independent isolates, *sensu stricto*, they are two individual strains. In contrast, if no distinguishing differences are seen between two isolates, then they are independent isolates of the same strain. These distinctions will increasingly impact the fields of molecular epidemiology and microbial forensics as today's technologies and their attendant discriminatory powers are utilized in such investigations.

## Authors' contributions

SAJ designed the study, performed the analysis and prepared the manuscript. IRP carried out the molecular experimental studies and assisted with the analysis and preparation of the manuscript. TB acquired part of the experimental data. JEL acquired the funding, supervised the project and revised the manuscript. TAC supervised the project and was critically involved in revising the manuscript and contributed essential intellectual content. All authors read and approved the manuscript.

## Supplementary Material

Additional File 1 **Gene differences matrix**. Number of gene differences based on strain-to-strain comparisons is shown. A "gene difference" is defined here as a 4-fold difference in the RMA-summarized probe set intensities. The cells are color-coded based on the number of gene differences using the scale below. Strains are ordered based on their relatedness as determined by hierarchical cluster analysis. Click here for file

Additional File 2 **Pearson correlation matrix**. R-Bioconductor was used to calculate Pearson correlation coefficients using RMA-summarized probe set intensities. The cells are color-coded to show relatedness and correlation (coefficient from 0-1) according to the scale below. Strains are ordered based on their relatedness as determined by hierarchical cluster analysis. Click here for file

Additional File 3 **Hierarchical Cluster Analysis**. RMA-summarized probe set intensities were used to hierarchically cluster (Euclidean means) all 207 isolates (top dendrogram) and all 10,208 genes (left dendrogram) in Spotfire. The heatmap shows RMA probe set intensities from low (green) to high (red). The top dendrogram is color-coded based on the 3 large clusters of *E. coli*. Click here for file

Additional File 4 **Conserved, core, backbone genes in *E. coli *and *Shigella***. Using the MAS 5.0 gene detection method, we filtered those probe sets that were called "present" in all 207 isolates. The 2256 conserved probe sets are listed here along with their gene description, when available. Click here for file

Additional File 5 **Conserved Intergenic Regions**. Using the MAS 5.0 gene detection method, we filtered those probe sets that were annotated as "intergenic" and called "present" in all 207 isolates. The 232 conserved intergenic probe sets are listed here along with their genome position and length. Click here for file
