# Supplementary figures and images for: Investigating the global genomic diversity of Escherichia coli using a multi-genome DNA microarray platform with novel gene prediction strategies
Source: BMC Genomics. 2011 Jul 6;12:349. doi: 10.1186/1471-2164-12-349 (PMC3146454; doi:10.1186/1471-2164-12-349)

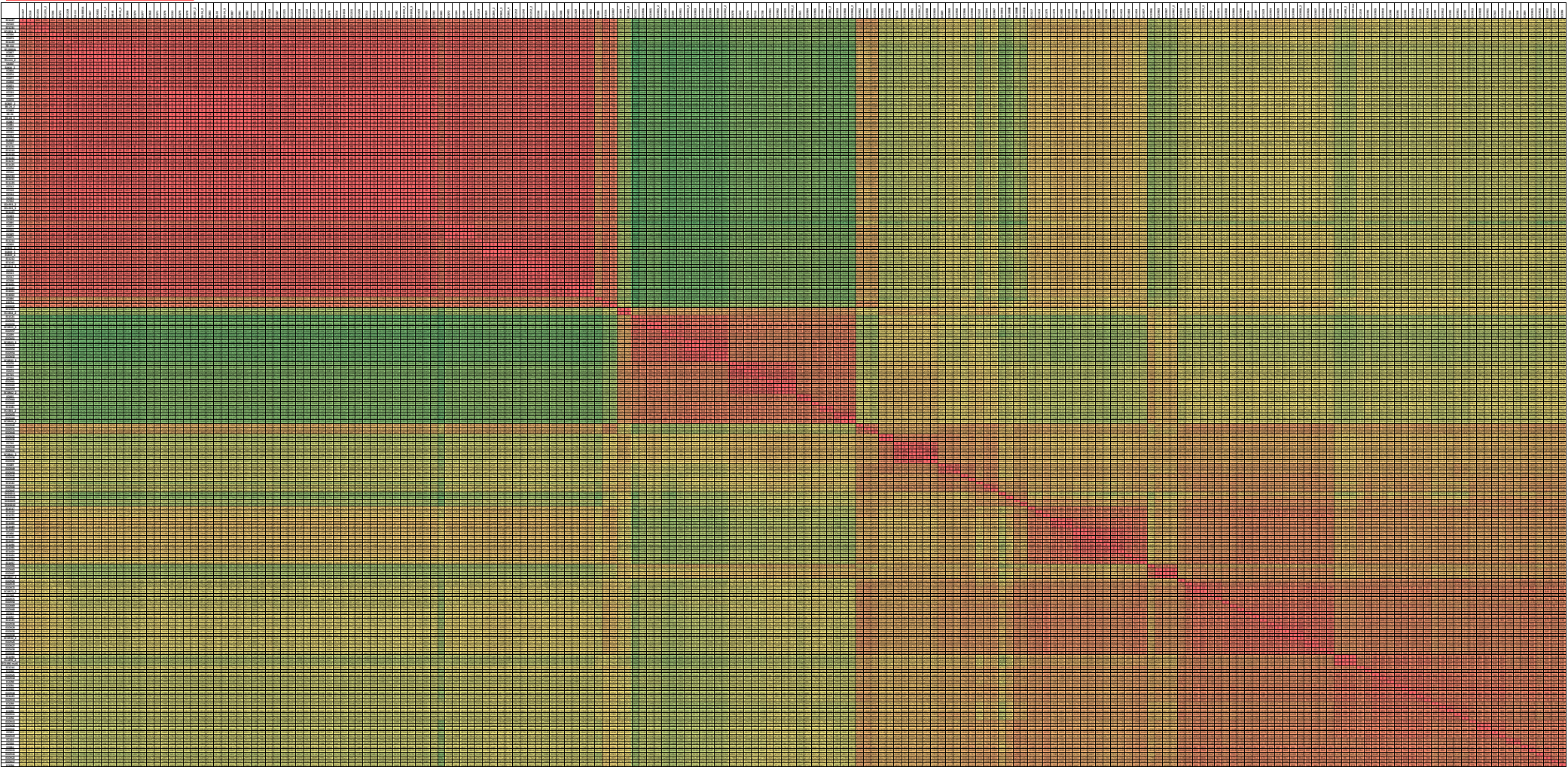

0

200

1000

2000

3000

4000

4890

Supplement: Additional File 1 — Gene differences matrix. Number of gene differences based on strain-to-strain comparisons is shown. A "gene difference" is defined here as a 4-fold difference in the RMA-summarized probe set intensities. The cells are color-coded based on the number of gene differences using the scale below. Strains are ordered based on their relatedness as determined by hierarchical cluster analysis. [file 1471-2164-12-349-S1.PDF]

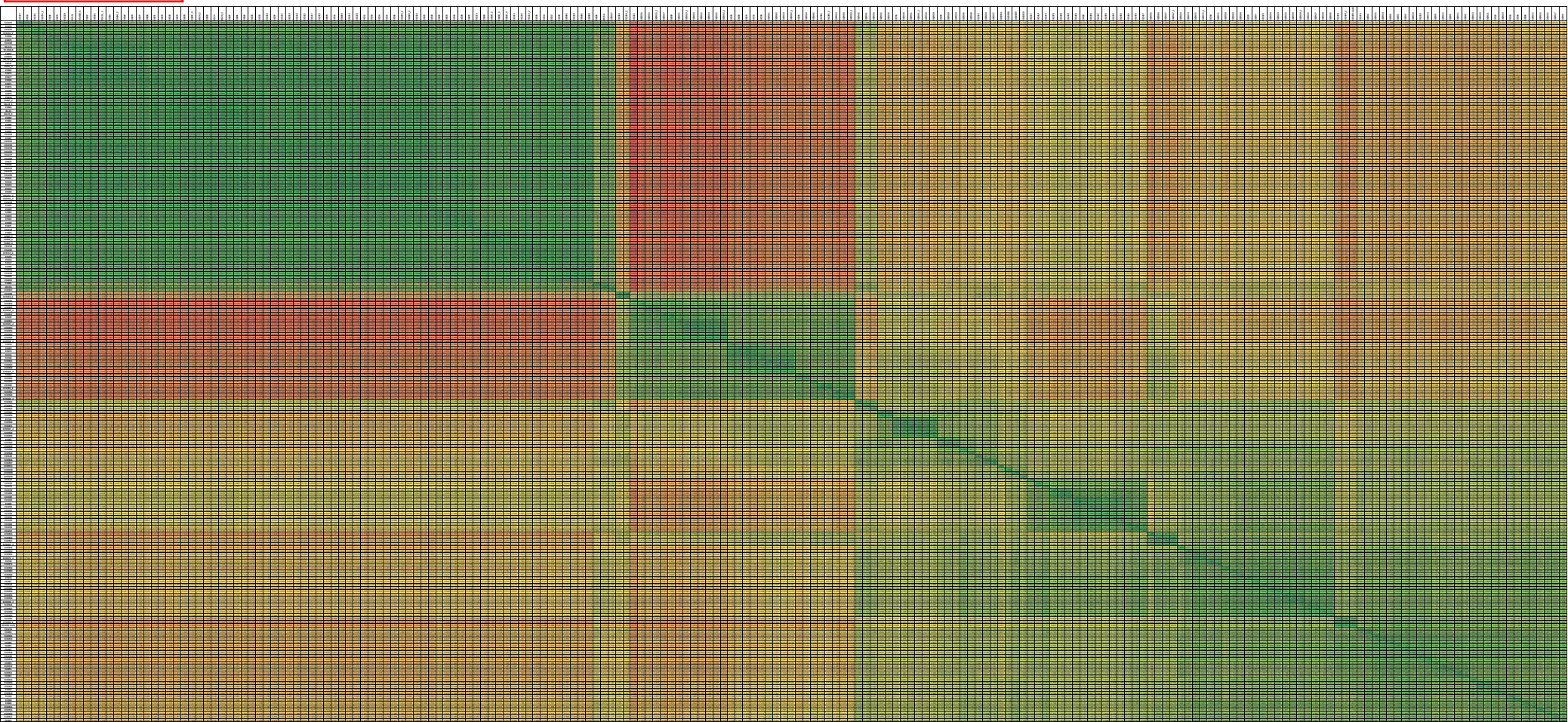

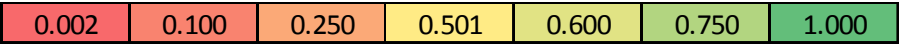

Supplement: Additional File 2 — Pearson correlation matrix. R-Bioconductor was used to calculate Pearson correlation coefficients using RMA-summarized probe set intensities. The cells are color-coded to show relatedness and correlation (coefficient from 0-1) according to the scale below. Strains are ordered based on their relatedness as determined by hierarchical cluster analysis. [file 1471-2164-12-349-S2.PDF]

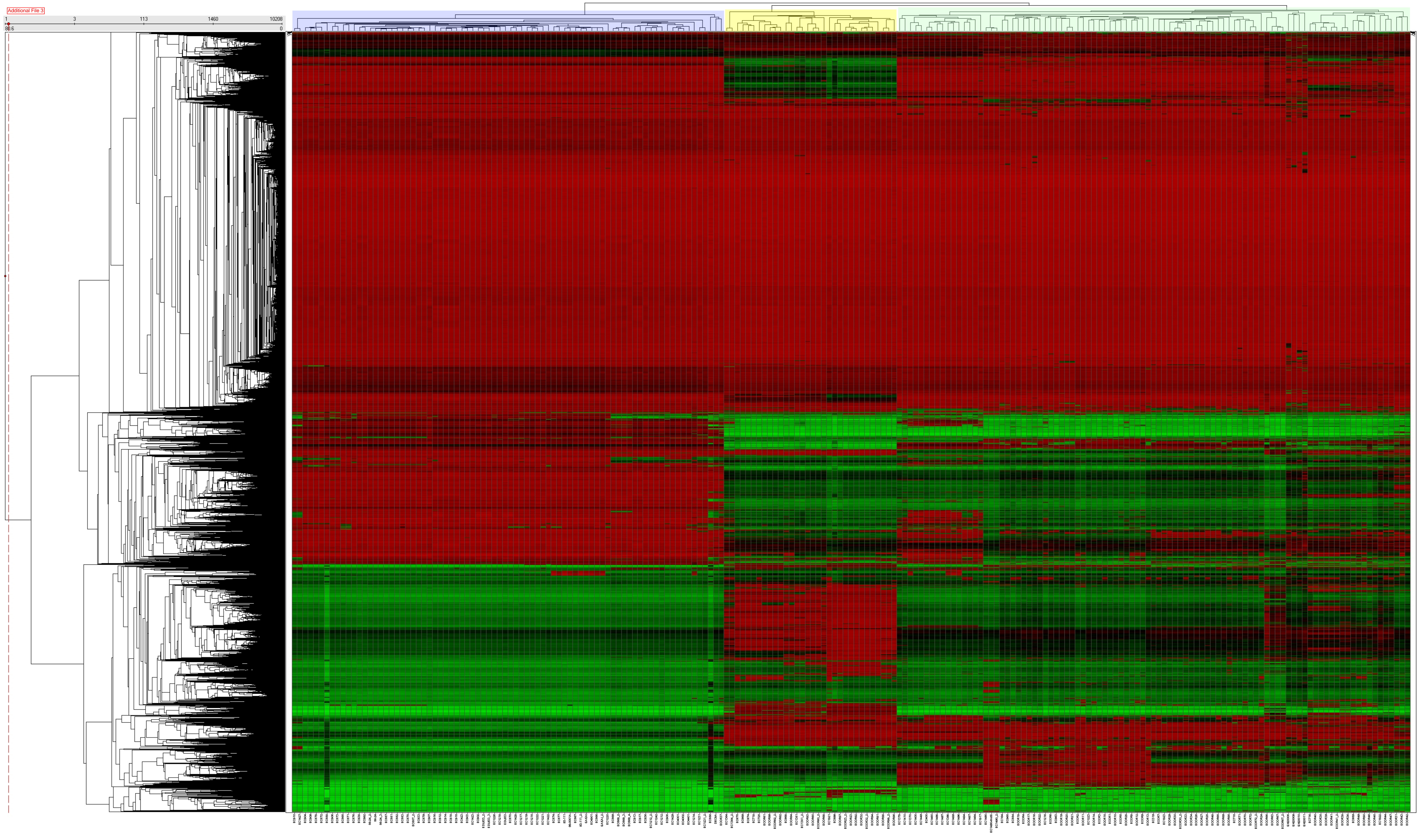

Supplement: Additional File 3 — Hierarchical Cluster Analysis. RMA-summarized probe set intensities were used to hierarchically cluster (Euclidean means) all 207 isolates (top dendrogram) and all 10,208 genes (left dendrogram) in Spotfire. The heatmap shows RMA probe set intensities from low (green) to high (red). The top dendrogram is color-coded based on the 3 large clusters of E. coli. [file 1471-2164-12-349-S3.PDF]
