# Supplementary material for: Investigating the global genomic diversity of Escherichia coli using a multi-genome DNA microarray platform with novel gene prediction strategies
Source: BMC Genomics. 2011 Jul 6;12:349. doi: 10.1186/1471-2164-12-349 (PMC3146454; doi:10.1186/1471-2164-12-349)
Supplement: Additional File 4 — Conserved, core, backbone genes in E. coli and Shigella. Using the MAS 5.0 gene detection method, we filtered those probe sets that were called "present" in all 207 isolates. The 2256 conserved probe sets are listed here along with their gene description, when available. [file 1471-2164-12-349-S4.PDF]

Additional File 4

ProbeSet ID	Genome	Locus ID	Gene
1759071_s_at	MG1655	b2503	NA
1759102_s_at	MG1655	b2567	rnc
1759105_s_at	MG1655	b4392	slt
1759106_s_at	MG1655	b3347	fkpA
1759108_s_at	MG1655	b1686	NA
1759109_s_at	SAKAI	ECs3063	NA
1759114_s_at	MG1655	b2214	yojL
1759122_s_at	MG1655	b0002	thrA
1759124_s_at	MG1655	b2138	yohG
1759127_s_at	MG1655	b3003	yghA
1759129_s_at	MG1655	b2958	yggN
1759130_s_at	MG1655	b1648	NA
1759134_s_at	MG1655	b1600	NA
1759162_at	CFT073	c5473	serB
1759164_s_at	MG1655	b2614	grpE
1759166_s_at	MG1655	b0191	yaeJ
1759170_s_at	MG1655	b4260	pepA
1759174_s_at	MG1655	b2384	NA
1759178_s_at	MG1655	b2169	fruB
1759181_s_at	MG1655	b1793	yoaF
1759182_s_at	MG1655	b3238	yhcN
1759185_s_at	MG1655	b0495	ybbA
1759186_s_at	CFT073	c0869	NA
1759188_s_at	EDL933	Z0138	yadG
1759191_s_at	MG1655	b3017	sufI
1759193_s_at	MG1655	b3351	yheR
1759201_s_at	MG1655	b3063	ygjE
1759202_s_at	MG1655	b1821	NA
1759212_s_at	MG1655	b0410	yajD
1759213_s_at	CFT073	c2225	NA
1759218_s_at	MG1655	b2168	fruK
1759219_s_at	MG1655	b4152	frdC
1759226_s_at	MG1655	b3201	yhbG
1759231_s_at	MG1655	b2897	ygfY
1759242_s_at	MG1655	b0630	lipB
1759257_s_at	CFT073	c0488	NA
1759265_s_at	MG1655	b0626	ybeM
1759287_s_at	MG1655	b1222	narX
1759291_s_at	MG1655	b0578	nfnB
1759293_s_at	MG1655	b0008	talB
1759299_s_at	MG1655	b0494	tesA
1759303_s_at	MG1655	b0624	crcB
1759304_s_at	EDL933	Z2024	NA
1759305_s_at	MG1655	b2602	yfiL
1759309_s_at	MG1655	b0812	dps
1759318_s_at	CFT073	c5343	NA
1759324_s_at	CFT073	c4442	NA
1759331_s_at	MG1655	b0960	NA
1759332_s_at	MG1655	b3182	dacB
1759333_s_at	MG1655	b4177	purA

1759339_at	MG1655	b4458	oxyS
1759343_s_at	CFT073	c3048	pepB
1759345_s_at	MG1655	b2765	ygcM
1759347_s_at	MG1655	b2900	yqfB
1759352_s_at	MG1655	b3313	rplP
1759362_s_at	CFT073	c3925	NA
1759368_s_at	MG1655	b3285	smf_2
1759376_s_at	MG1655	b3858	yihD
1759379_s_at	MG1655	b3860	dsbA
1759384_s_at	MG1655	b4398	creB
1759393_s_at	MG1655	b2527	NA
1759395_s_at	MG1655	b3544	dppA
1759399_s_at	MG1655	b2593	yfiH
1759400_s_at	MG1655	b2123	yehR
1759408_s_at	MG1655	b0825	NA
1759421_s_at	MG1655	b1641	slyB
1759428_s_at	MG1655	b2195	NA
1759429_s_at	MG1655	b3409	feoB
1759432_s_at	MG1655	b0452	tesB
1759434_s_at	MG1655	b0078	ilvH
1759438_s_at	MG1655	b2288	nuoA
1759439_at	MG1655	b4451	ryhB
1759445_s_at	MG1655	b2997	NA
1759459_s_at	MG1655	b3031	yqiA
1759467_s_at	MG1655	b1679	NA
1759477_s_at	EDL933	Z3503	NA
1759485_s_at	MG1655	b3395	yrfD
1759490_s_at	MG1655	b1237	hns
1759494_s_at	MG1655	b1112	ycfR
1759496_s_at	MG1655	b3156	yhbS
1759501_s_at	EDL933	Z3235	NA
1759504_s_at	EDL933	Z3516	NA
1759509_s_at	MG1655	b3004	NA
1759510_s_at	MG1655	b3699	gyrB
1759511_s_at	MG1655	b2555	yfhG
1759512_s_at	MG1655	b1707	NA
1759515_s_at	MG1655	b3431	glgX
1759520_s_at	MG1655	b3746	yieN
1759522_s_at	CFT073	c3214	NA
1759529_s_at	MG1655	b2495	NA
1759531_s_at	MG1655	b4406	yaeP
1759538_s_at	MG1655	b1186	nhaB
1759542_s_at	MG1655	b0902	pflA
1759546_s_at	MG1655	b1610	tus
1759549_s_at	MG1655	b0711	ybgJ
1759552_s_at	MG1655	b3248	yhdE
1759563_s_at	MG1655	b3859	yihE
1759565_s_at	MG1655	b3243	yhcS
1759569_s_at	MG1655	b2828	lgt
1759573_s_at	MG1655	b0168	map
1759574_s_at	MG1655	b0660	ybeZ

1759586_at	CFT073	c4605	NA
1759588_s_at	MG1655	b3464	ftsY
1759591_s_at	EDL933	Z1038	NA
1759596_s_at	CFT073	c2169	NA
1759598_s_at	MG1655	b0832	NA
1759599_s_at	MG1655	b1126	potA
1759606_s_at	MG1655	b3856	mobB
1759609_s_at	MG1655	b0239	yafA
1759611_s_at	MG1655	b1693	aroD
1759615_s_at	MG1655	b0086	murF
1759618_s_at	MG1655	b1295	ymjA
1759624_s_at	MG1655	b0579	ybdF
1759628_s_at	EDL933	Z5349	metR
1759630_s_at	CFT073	c0693	NA
1759645_s_at	MG1655	b1912	pgsA
1759648_s_at	MG1655	b3734	atpA
1759664_s_at	MG1655	b1097	yceG
1759666_s_at	MG1655	b3807	cyaY
1759675_s_at	MG1655	b0097	yacA
1759683_s_at	MG1655	b3279	yrdA
1759685_s_at	MG1655	b1901	araF
1759694_s_at	MG1655	b3985	rplJ
1759697_s_at	MG1655	b1452	NA
1759699_s_at	MG1655	b2687	ygaG
1759701_s_at	EDL933	Z1299	uup
1759704_s_at	MG1655	b2530	iscS
1759706_s_at	MG1655	b2079	baeR
1759712_at	SAKAI	ECs4876	NA
1759715_s_at	MG1655	b0970	yccA
1759719_s_at	CFT073	c0799	NA
1759721_s_at	MG1655	b1820	NA
1759726_s_at	MG1655	b3118	tdcA
1759729_s_at	MG1655	b0639	NA
1759732_s_at	MG1655	b2378	ddg
1759736_s_at	MG1655	b3887	NA
1759740_s_at	MG1655	b0485	ybaS
1759744_s_at	CFT073	c0872	ybhO
1759745_at	CFT073	c4437	NA
1759748_s_at	MG1655	b0737	tolQ
1759749_s_at	MG1655	b0897	ycaC
1759758_s_at	MG1655	b3548	yhjY
1759761_s_at	SAKAI	ECs2304	NA
1759770_s_at	MG1655	b0659	ybeY
1759776_s_at	MG1655	b2477	nlpB
1759780_s_at	MG1655	b1677	lpp
1759782_s_at	MG1655	b4353	yjiX
1759784_s_at	CFT073	c1008	NA
1759788_s_at	MG1655	b3927	glpF
1759789_s_at	CFT073	c3095	rseB
1759795_s_at	CFT073	c2375	NA
1759798_s_at	MG1655	b2604	yfiN

1759799_s_at	MG1655	b1281	pyrF
1759801_s_at	MG1655	b0755	gpmA
1759803_s_at	MG1655	b2340	NA
1759812_s_at	MG1655	b1858	NA
1759817_s_at	MG1655	b3949	frwC
1759834_s_at	MG1655	b1899	araH_1
1759837_s_at	MG1655	b1037	csgG
1759841_s_at	CFT073	c2676	NA
1759847_s_at	MG1655	b2615	yfjB
1759853_s_at	MG1655	b0925	ycbB
1759857_s_at	MG1655	b3747	kup
1759858_x_at	CFT073	c1062	NA
1759861_s_at	MG1655	b0090	murG
1759865_s_at	MG1655	b3737	atpE
1759867_s_at	MG1655	b3999	yjaG
1759872_s_at	CFT073	c5225	NA
1759873_s_at	MG1655	b1848	yebG
1759880_s_at	MG1655	b4020	yjbB
1759887_s_at	MG1655	b2700	ygaD
1759892_s_at	MG1655	b2906	visC
1759898_s_at	MG1655	b1863	ruvC
1759900_s_at	CFT073	c2122	NA
1759901_s_at	CFT073	c1516	NA
1759904_s_at	MG1655	b3036	ygiA
1759906_s_at	MG1655	b1189	dadA
1759909_s_at	MG1655	b2080	NA
1759911_s_at	MG1655	b3451	ugpE
1759918_s_at	MG1655	b0882	clpA
1759921_s_at	MG1655	b3345	yheN
1759922_s_at	MG1655	b0087	mraY
1759927_s_at	MG1655	b4055	aphA
1759929_s_at	MG1655	b3286	smf_1
1759933_s_at	MG1655	b3147	yraM
1759937_s_at	MG1655	b4229	ytfS
1759939_s_at	MG1655	b0032	carA
1759940_s_at	MG1655	b2478	dapA
1759946_s_at	MG1655	b3096	yqjB
1759951_s_at	CFT073	c4360	NA
1759977_s_at	MG1655	b3356	yhfA
1759981_s_at	MG1655	b1330	NA
1759987_s_at	MG1655	b3056	cca
1759989_s_at	MG1655	b3150	yraP
1759996_s_at	MG1655	b1650	nemA
1759997_s_at	CFT073	c3917	NA
1759998_s_at	MG1655	b3824	NA
1760016_s_at	MG1655	b1902	yecI
1760019_s_at	CFT073	c1714	NA
1760021_s_at	MG1655	b4223	NA
1760022_s_at	CFT073	c1051	NA
1760025_s_at	CFT073	c0182	NA
1760028_s_at	CFT073	c2748	NA

1760031_s_at	MG1655	b1103	ycfF
1760033_s_at	MG1655	b1727	yniC
1760038_s_at	EDL933	Z3676	NA
1760048_s_at	MG1655	b0774	bioA
1760052_s_at	MG1655	b0178	hlpA
1760057_s_at	MG1655	b4144	yjeI
1760058_s_at	MG1655	b3763	pssR
1760060_s_at	MG1655	b3452	ugpA
1760064_s_at	MG1655	b0521	arcC
1760085_at	CFT073	c5290	NA
1760094_s_at	MG1655	b0950	pqiA
1760096_s_at	CFT073	c4113	NA
1760097_s_at	MG1655	b0921	smtA
1760113_s_at	MG1655	b1744	NA
1760121_s_at	MG1655	b2991	hybF
1760128_s_at	MG1655	b0422	xseB
1760130_s_at	MG1655	b2142	yohK
1760134_s_at	MG1655	b3249	mreD
1760138_s_at	MG1655	b2199	ccmC
1760139_s_at	CFT073	c2541	NA
1760140_s_at	MG1655	b4404	yifM_1
1760147_s_at	MG1655	b2282	nuoH
1760161_s_at	MG1655	b0393	yaiD
1760166_s_at	CFT073	c4088	NA
1760176_s_at	MG1655	b1870	yecO
1760182_s_at	MG1655	b2599	pheA
1760186_s_at	MG1655	b0820	ybiT
1760189_s_at	MG1655	b1791	yeaN
1760190_s_at	MG1655	b4188	yjfN
1760193_s_at	MG1655	b4042	dgkA
1760197_s_at	MG1655	b2610	ffh
1760202_s_at	MG1655	b2505	NA
1760205_s_at	MG1655	b4174	hflK
1760223_s_at	MG1655	b3106	yhaK
1760225_s_at	MG1655	b3987	rpoB
1760226_s_at	MG1655	b2832	NA
1760237_s_at	MG1655	b2144	sanA
1760241_s_at	MG1655	b4200	rpsF
1760242_s_at	MG1655	b3359	argD
1760252_s_at	MG1655	b3398	yrfF
1760255_s_at	MG1655	b1287	yciW
1760257_s_at	MG1655	b1703	ydiA
1760263_s_at	MG1655	b0182	lpxB
1760266_at	CFT073	c5012	NA
1760270_s_at	MG1655	b2207	napD
1760273_s_at	MG1655	b2432	NA
1760274_s_at	MG1655	b3592	yibF
1760275_s_at	MG1655	b3008	metC
1760290_s_at	MG1655	b0727	sucB
1760294_s_at	MG1655	b0075	leuL
1760299_s_at	MG1655	b3360	pabA

1760302_s_at	MG1655	b3529	yhjK
1760303_s_at	MG1655	b1903	NA
1760316_s_at	MG1655	b0120	speD
1760319_s_at	CFT073	c0643	ybcI
1760326_s_at	MG1655	b3183	yhbZ
1760339_s_at	MG1655	b4151	frdD
1760343_s_at	SAKAI	ECs5595	NA
1760355_s_at	MG1655	b2276	nuoN
1760362_s_at	MG1655	b1329	NA
1760363_s_at	MG1655	b1702	ppsA
1760364_s_at	MG1655	b3540	dppF
1760368_s_at	MG1655	b4155	yjeA
1760378_s_at	MG1655	b4382	deoA
1760380_s_at	MG1655	b3839	NA
1760387_s_at	MG1655	b3745	yieM
1760388_s_at	MG1655	b1928	yedD
1760391_s_at	CFT073	c5361	NA
1760394_s_at	MG1655	b1594	mlc
1760399_s_at	CFT073	c2316	NA
1760411_s_at	MG1655	b1683	NA
1760412_s_at	CFT073	c0476	tauD
1760413_s_at	MG1655	b2994	hybC
1760423_s_at	MG1655	b1780	yeaD
1760426_s_at	MG1655	b4002	NA
1760432_s_at	MG1655	b3666	uhpT
1760437_s_at	CFT073	c4168	NA
1760452_s_at	MG1655	b2681	NA
1760453_s_at	MG1655	b1921	fliZ
1760457_s_at	MG1655	b0407	yajC
1760461_s_at	CFT073	c0828	NA
1760462_s_at	EDL933	Z1278	asnS
1760468_s_at	MG1655	b1624	NA
1760469_s_at	CFT073	c0784	NA
1760489_s_at	MG1655	b4138	dcuA
1760490_x_at	MG1655	b4421	ldrB
1760491_s_at	MG1655	b2081	yegQ
1760498_s_at	MG1655	b3247	NA
1760501_s_at	MG1655	b2197	ccmE
1760502_s_at	MG1655	b1859	NA
1760512_s_at	MG1655	b2909	ygfB
1760515_s_at	MG1655	b2792	NA
1760518_s_at	MG1655	b0997	torA
1760524_s_at	MG1655	b3795	yifK
1760527_s_at	MG1655	b2595	NA
1760533_s_at	EDL933	Z2450	ycjG
1760534_s_at	MG1655	b3786	wecB
1760535_s_at	MG1655	b2205	napG
1760539_s_at	MG1655	b3208	mtgA
1760540_s_at	MG1655	b2515	NA
1760543_s_at	MG1655	b4362	dnaT
1760544_s_at	MG1655	b4373	rimI

1760554_s_at	MG1655	b0585	fes
1760555_s_at	MG1655	b3117	tdcB
1760560_s_at	MG1655	b0185	accA
1760576_s_at	CFT073	c3759	NA
1760584_s_at	MG1655	b2137	yohF
1760587_s_at	CFT073	c2046	NA
1760597_s_at	MG1655	b1066	rimJ
1760600_s_at	MG1655	b1539	ydfG
1760601_s_at	MG1655	b0155	yadQ
1760604_s_at	CFT073	c0877	ybhF
1760610_s_at	CFT073	c4188	yhgA
1760617_s_at	CFT073	c4819	glnA
1760618_s_at	MG1655	b1308	pspE
1760619_s_at	MG1655	b1622	malY
1760622_s_at	CFT073	c2147	NA
1760630_s_at	MG1655	b3432	glgB
1760634_s_at	MG1655	b4063	soxR
1760637_s_at	MG1655	b1187	fadR
1760640_at	SAKAI	ECs0702	NA
1760643_s_at	MG1655	b1075	flgD
1760646_s_at	MG1655	b3186	rplU
1760649_s_at	MG1655	b4026	yjbE
1760652_s_at	MG1655	b4384	deoD
1760653_s_at	MG1655	b0475	hemH
1760656_s_at	MG1655	b0968	NA
1760665_s_at	CFT073	c3040	NA
1760671_s_at	MG1655	b2573	rpoE
1760674_s_at	MG1655	b2565	recO
1760678_s_at	CFT073	c3549	NA
1760679_at	MG1655	b4459	ryjA
1760682_s_at	MG1655	b3730	glmU
1760687_s_at	CFT073	c5274	yjfC
1760690_s_at	MG1655	b1099	holB
1760691_s_at	MG1655	b3203	yhbH
1760701_s_at	MG1655	b1254	ispZ
1760716_s_at	MG1655	b3123	rnpB
1760724_s_at	MG1655	b2266	elaB
1760725_s_at	MG1655	b3343	yheL
1760738_s_at	MG1655	b3113	NA
1760745_s_at	MG1655	b3342	rpsL
1760748_s_at	MG1655	b4135	yjdC
1760752_s_at	MG1655	b3317	rplB
1760754_s_at	MG1655	b2617	smpA
1760755_s_at	CFT073	c0897	NA
1760762_s_at	MG1655	b1627	NA
1760763_s_at	MG1655	b0175	cdsA
1760766_s_at	MG1655	b1528	NA
1760773_s_at	MG1655	b3310	rplN
1760779_s_at	CFT073	c0545	NA
1760781_s_at	MG1655	b3110	yhaO
1760785_s_at	MG1655	b3675	yidG

1760786_s_at	MG1655	b2581	yfiF
1760787_s_at	EDL933	Z1714	flgE
1760792_s_at	SAKAI	ECs5487	NA
1760796_s_at	MG1655	b3414	yhgI
1760800_s_at	EDL933	Z1030	ybiO
1760802_s_at	MG1655	b4198	sgaE
1760803_s_at	MG1655	b0758	galT
1760808_s_at	MG1655	b4405	yifM_2
1760813_s_at	MG1655	b1864	yebC
1760815_s_at	MG1655	b3676	yidH
1760820_s_at	MG1655	b1835	yebU
1760828_s_at	MG1655	b0080	fruR
1760837_s_at	CFT073	c2782	glpA
1760840_s_at	CFT073	c0159	yadE
1760844_s_at	MG1655	b0877	ybjX
1760848_s_at	MG1655	b1098	tmk
1760855_x_at	MG1655	b4420	rdlA
1760866_s_at	CFT073	c1718	NA
1760874_s_at	MG1655	b0435	bolA
1760881_s_at	MG1655	b1276	acnA
1760884_s_at	CFT073	c4064	NA
1760890_s_at	MG1655	b2256	NA
1760891_s_at	MG1655	b1476	fdnI
1760892_s_at	SAKAI	ECs0126	NA
1760894_s_at	MG1655	b1184	umuC
1760898_s_at	MG1655	b0089	ftsW
1760899_s_at	EDL933	Z1046	NA
1760901_s_at	CFT073	c4608	yidQ
1760904_s_at	MG1655	b3181	greA
1760908_s_at	MG1655	b3204	ptsN
1760909_s_at	CFT073	c1905	NA
1760911_s_at	MG1655	b1056	yceI
1760914_s_at	CFT073	c4376	NA
1760925_s_at	MG1655	b2558	yfhD
1760929_s_at	MG1655	b3355	prkB
1760932_s_at	MG1655	b1605	NA
1760934_s_at	SAKAI	ECs3824	NA
1760938_s_at	MG1655	b1531	marA
1760940_s_at	EDL933	Z3731	NA
1760943_s_at	MG1655	b4025	pgi
1760946_s_at	MG1655	b1640	NA
1760950_s_at	MG1655	b0833	NA
1760952_s_at	CFT073	c3248	NA
1760954_s_at	MG1655	b2280	nuoJ
1760956_s_at	MG1655	b3233	yhcB
1760958_s_at	MG1655	b3892	fdoI
1760965_s_at	MG1655	b1829	htpX
1760967_s_at	MG1655	b1057	NA
1760971_s_at	MG1655	b3749	rbsA
1760975_s_at	MG1655	b4395	gpmB
1760978_s_at	MG1655	b0831	NA

1760982_s_at	MG1655	b3499	yhiR
1760985_s_at	CFT073	c5018	yjbN
1760988_s_at	MG1655	b3861	yihF
1760991_s_at	MG1655	b4176	yjeT
1761007_s_at	MG1655	b0962	helD
1761022_s_at	MG1655	b1906	yecH
1761025_s_at	MG1655	b2572	rseA
1761031_s_at	MG1655	b2890	lysS
1761043_s_at	MG1655	b4187	aidB
1761055_s_at	EDL933	Z2172	ydeB
1761057_s_at	MG1655	b2965	speC
1761063_s_at	CFT073	c2858	NA
1761066_s_at	MG1655	b3433	asd
1761071_s_at	MG1655	b3523	yhjE
1761073_s_at	MG1655	b3040	ygiE
1761076_s_at	MG1655	b0384	psiF
1761079_s_at	MG1655	b0380	NA
1761080_x_at	MG1655	b2598	pheL
1761093_s_at	MG1655	b0158	yadT
1761096_s_at	MG1655	b3619	rfaD
1761097_s_at	MG1655	b1961	dcm
1761099_s_at	MG1655	b3754	yieO
1761103_s_at	MG1655	b2101	yegW
1761110_s_at	MG1655	b1675	NA
1761114_s_at	MG1655	b3556	cspA
1761130_s_at	MG1655	b0888	trxB
1761135_s_at	MG1655	b2471	yffB
1761136_s_at	MG1655	b0872	NA
1761139_s_at	MG1655	b2236	yfaE
1761143_s_at	MG1655	b1874	cutC
1761152_s_at	MG1655	b2525	fdx
1761153_s_at	MG1655	b3055	ygiM
1761155_at	SAKAI	ECs2890	NA
1761163_s_at	MG1655	b2512	NA
1761165_s_at	MG1655	b3205	yhbJ
1761168_s_at	MG1655	b3711	yidZ
1761169_s_at	MG1655	b0004	thrC
1761171_s_at	MG1655	b0922	mukF
1761178_s_at	MG1655	b2421	cysM
1761188_s_at	MG1655	b2664	ygaE
1761194_s_at	MG1655	b0791	ybhQ
1761198_s_at	MG1655	b1840	NA
1761206_s_at	MG1655	b2202	napC
1761210_s_at	MG1655	b1238	tdk
1761214_s_at	MG1655	b3241	yhcQ
1761220_s_at	MG1655	b4194	sgaB
1761243_s_at	MG1655	b2535	csiE
1761244_s_at	MG1655	b3315	rplV
1761248_s_at	MG1655	b4175	hflC
1761260_s_at	MG1655	b2468	NA
1761262_s_at	MG1655	b2292	yfbS

1761274_at	MG1655	b4426	IS061
1761277_s_at	MG1655	b1194	ycgR
1761294_s_at	EDL933	Z1052	NA
1761295_s_at	EDL933	Z0555	ybaO
1761297_s_at	MG1655	b0817	NA
1761299_s_at	CFT073	c3309	NA
1761300_s_at	MG1655	b3983	rplK
1761305_s_at	MG1655	b0635	mrdA
1761307_s_at	MG1655	b2289	lrhA
1761322_s_at	MG1655	b0243	proA
1761326_s_at	MG1655	b3470	NA
1761327_s_at	CFT073	c1016	NA
1761330_s_at	MG1655	b2522	sseB
1761334_s_at	MG1655	b2585	pssA
1761337_s_at	CFT073	c4081	NA
1761339_s_at	MG1655	b0099	mutT
1761352_s_at	CFT073	c4868	NA
1761354_s_at	SAKAI	ECs5443	NA
1761355_s_at	MG1655	b2124	yehS
1761359_s_at	MG1655	b2323	fabB
1761361_s_at	MG1655	b1053	yceE
1761362_s_at	MG1655	b1232	purU
1761379_s_at	MG1655	b3111	NA
1761380_s_at	MG1655	b2426	ucpA
1761385_s_at	MG1655	b2436	hemF
1761388_s_at	CFT073	c0855	bioB
1761399_s_at	MG1655	b1601	NA
1761402_s_at	MG1655	b3982	nusG
1761406_s_at	MG1655	b1748	NA
1761410_s_at	MG1655	b1873	NA
1761413_s_at	CFT073	c2255	NA
1761420_s_at	MG1655	b3429	glgA
1761422_s_at	MG1655	b3400	NA
1761428_s_at	MG1655	b0009	mog
1761432_s_at	MG1655	b0464	acrR
1761433_s_at	MG1655	b3405	ompR
1761436_s_at	MG1655	b0162	NA
1761437_s_at	CFT073	c0141	NA
1761440_s_at	MG1655	b1595	ynfL
1761441_s_at	MG1655	b1269	yciL
1761449_s_at	MG1655	b1642	slyA
1761453_s_at	MG1655	b0773	ybhB
1761455_s_at	MG1655	b2618	NA
1761463_s_at	MG1655	b1788	NA
1761471_s_at	CFT073	c1312	NA
1761476_s_at	MG1655	b2750	cysC
1761484_s_at	EDL933	Z2547	trpE_2
1761485_s_at	MG1655	b3836	NA
1761486_s_at	MG1655	b1623	add
1761487_s_at	MG1655	b3018	plsC
1761492_s_at	MG1655	b1712	himA

1761493_s_at	MG1655	b2411	NA
1761494_s_at	MG1655	b3939	metB
1761499_s_at	MG1655	b2139	yohH
1761502_s_at	MG1655	b1193	mltE
1761505_s_at	MG1655	b3702	dnaA
1761513_s_at	CFT073	c1103	NA
1761516_s_at	CFT073	c0503	NA
1761522_s_at	MG1655	b0170	tsf
1761524_s_at	MG1655	b3657	yicJ
1761526_s_at	MG1655	b2263	yfbB
1761528_s_at	MG1655	b4062	soxS
1761540_s_at	MG1655	b4401	arcA
1761541_s_at	MG1655	b2126	yehU
1761543_s_at	MG1655	b2200	ccmB
1761544_s_at	MG1655	b1286	rnb
1761550_s_at	MG1655	b2895	fldB
1761554_s_at	MG1655	b2497	uraA
1761571_s_at	MG1655	b2204	napH
1761574_s_at	MG1655	b3967	murI
1761575_s_at	MG1655	b2667	NA
1761577_s_at	MG1655	b2960	yggH
1761589_s_at	MG1655	b4054	tyrB
1761600_s_at	MG1655	b3780	rhlB
1761604_s_at	MG1655	b0963	mgsA
1761605_s_at	MG1655	b3560	glyQ
1761606_s_at	MG1655	b1958	yedI
1761614_s_at	CFT073	c0205	NA
1761620_s_at	MG1655	b4240	treB
1761626_s_at	CFT073	c4993	NA
1761628_s_at	MG1655	b1130	phoP
1761629_s_at	MG1655	b2413	cysZ
1761635_s_at	MG1655	b1651	gloA
1761637_s_at	MG1655	b3163	NA
1761641_s_at	MG1655	b0736	ybgC
1761642_s_at	MG1655	b3259	prmA
1761644_s_at	MG1655	b3673	emrD
1761649_s_at	MG1655	b1226	narJ
1761655_s_at	MG1655	b4386	lplA
1761660_s_at	CFT073	c1353	rne
1761663_s_at	MG1655	b3863	polA
1761664_s_at	MG1655	b0114	aceE
1761667_s_at	MG1655	b3435	gntU_2
1761668_s_at	MG1655	b2301	yfcF
1761670_s_at	MG1655	b2670	NA
1761676_s_at	CFT073	c3343	NA
1761680_s_at	CFT073	c0723	NA
1761682_s_at	MG1655	b3913	NA
1761684_s_at	MG1655	b0472	recR
1761685_s_at	MG1655	b2398	yfeC
1761688_s_at	EDL933	Z2646	ydgR
1761701_s_at	MG1655	b2174	NA

1761705_s_at	MG1655	b0904	focA
1761707_s_at	MG1655	b3741	gidA
1761715_s_at	CFT073	c3384	mltA
1761717_s_at	MG1655	b3234	degQ
1761724_s_at	MG1655	b0655	NA
1761726_s_at	MG1655	b1244	oppB
1761729_s_at	MG1655	b1104	ycfL
1761738_s_at	EDL933	Z2534	NA
1761740_s_at	MG1655	b0151	fhuC
1761748_s_at	MG1655	b0751	pnuC
1761758_s_at	MG1655	b3739	atpI
1761759_s_at	MG1655	b2287	nuoB
1761763_s_at	MG1655	b2616	recN
1761764_s_at	MG1655	b1929	yedE
1761767_s_at	MG1655	b0853	ybjN
1761768_s_at	MG1655	b0967	NA
1761771_s_at	MG1655	b1192	NA
1761775_s_at	MG1655	b3387	dam
1761776_s_at	MG1655	b1447	NA
1761777_s_at	MG1655	b1768	NA
1761783_s_at	MG1655	b1176	minC
1761790_s_at	MG1655	b0200	gmhB
1761794_s_at	MG1655	b1033	ycdW
1761800_s_at	CFT073	c4139	yhfC
1761801_s_at	CFT073	c0788	ybgH
1761804_s_at	MG1655	b0658	ybeX
1761806_s_at	MG1655	b3401	NA
1761815_s_at	MG1655	b0231	dinB
1761816_s_at	CFT073	c1624	NA
1761817_s_at	CFT073	c4661	NA
1761819_s_at	MG1655	b0473	htpG
1761832_s_at	CFT073	c0904	ybiR
1761834_s_at	MG1655	b0999	yccD
1761839_s_at	MG1655	b3539	yhjV
1761844_s_at	MG1655	b0166	dapD
1761845_s_at	MG1655	b3167	rbfA
1761847_s_at	MG1655	b2838	lysA
1761851_s_at	MG1655	b4212	ytfH
1761856_s_at	MG1655	b3316	rpsS
1761865_s_at	MG1655	b0444	ybaX
1761866_s_at	MG1655	b0597	ybdB
1761872_s_at	MG1655	b3986	rplL
1761879_s_at	CFT073	c3526	NA
1761898_s_at	MG1655	b0640	holA
1761899_s_at	MG1655	b0105	NA
1761907_s_at	MG1655	b2786	barA
1761908_s_at	CFT073	c0670	NA
1761910_s_at	CFT073	c2728	NA
1761915_s_at	MG1655	b1046	ymdC
1761916_s_at	CFT073	c0485	NA
1761917_s_at	MG1655	b3319	rplD

1761920_s_at	CFT073	c3983	NA
1761921_s_at	MG1655	b0490	ybbL
1761924_s_at	MG1655	b3126	garL
1761925_s_at	MG1655	b1812	pabB
1761926_s_at	CFT073	c4010	NA
1761931_s_at	MG1655	b0952	ymbA
1761932_s_at	MG1655	b3187	ispB
1761937_s_at	MG1655	b4179	NA
1761941_s_at	MG1655	b1045	NA
1761944_s_at	CFT073	c5458	NA
1761950_s_at	MG1655	b1713	pheT
1761958_s_at	MG1655	b0677	nagA
1761962_s_at	MG1655	b0759	galE
1761964_s_at	MG1655	b2582	trxC
1761969_s_at	MG1655	b1898	araH_2
1761998_s_at	MG1655	b3242	yhcR
1761999_s_at	MG1655	b3340	fusA
1762003_s_at	MG1655	b3207	yrbL
1762031_s_at	MG1655	b3921	yiiR
1762036_s_at	MG1655	b3936	rpmE
1762037_s_at	MG1655	b3424	glpG
1762047_s_at	MG1655	b4365	yjjQ
1762051_s_at	CFT073	c2992	NA
1762053_s_at	MG1655	b3192	yrbC
1762058_s_at	EDL933	Z4708	NA
1762061_s_at	MG1655	b0802	ybiJ
1762074_s_at	MG1655	b3463	ftsE
1762075_s_at	MG1655	b3312	rpmC
1762085_s_at	MG1655	b2346	vacJ
1762099_s_at	MG1655	b0678	nagB
1762100_s_at	MG1655	b3394	yrfC
1762105_s_at	MG1655	b1073	flgB
1762106_s_at	MG1655	b0788	ybhN
1762109_s_at	MG1655	b0189	NA
1762117_s_at	MG1655	b3367	nirC
1762122_s_at	MG1655	b0977	hyaF
1762130_s_at	MG1655	b1207	prsA
1762132_s_at	MG1655	b0740	tolB
1762133_s_at	MG1655	b0074	leuA
1762140_s_at	MG1655	b0181	lpxA
1762143_s_at	MG1655	b3766	ilvL
1762145_s_at	MG1655	b0023	rpsT
1762151_s_at	MG1655	b1926	fliT
1762154_at	MG1655	b4417	rybB
1762158_s_at	MG1655	b0402	proY
1762166_s_at	MG1655	b4393	trpR
1762171_s_at	CFT073	c4253	livJ
1762176_s_at	CFT073	c0864	NA
1762177_s_at	MG1655	b4013	metA
1762180_s_at	EDL933	Z3144	NA
1762182_s_at	MG1655	b3339	tufA

1762184_s_at	MG1655	b3845	fadA
1762187_s_at	MG1655	b1631	NA
1762196_s_at	MG1655	b0173	NA
1762198_s_at	MG1655	b2312	purF
1762205_s_at	MG1655	b2476	purC
1762208_s_at	MG1655	b1038	csgF
1762214_s_at	MG1655	b3231	rplM
1762221_s_at	MG1655	b0399	phoB
1762224_s_at	MG1655	b3384	trpS
1762236_s_at	MG1655	b0906	ycaP
1762238_s_at	MG1655	b1294	sapA
1762240_s_at	MG1655	b3931	hslU
1762241_s_at	CFT073	c4362	NA
1762242_s_at	CFT073	c1129	NA
1762255_s_at	MG1655	b3304	rplR
1762258_s_at	MG1655	b1905	ftn
1762265_s_at	MG1655	b4180	yjfH
1762271_s_at	MG1655	b3667	uhpC
1762272_s_at	CFT073	c2717	yejF
1762274_s_at	MG1655	b3923	yiiT
1762275_s_at	MG1655	b3009	yghB
1762286_s_at	MG1655	b3634	NA
1762287_s_at	MG1655	b2380	NA
1762291_s_at	MG1655	b1473	yddG
1762294_s_at	MG1655	b2966	yqgA
1762298_s_at	MG1655	b2534	NA
1762300_s_at	MG1655	b0196	rcsF
1762305_s_at	MG1655	b4202	rpsR
1762306_s_at	MG1655	b3210	arcB
1762309_s_at	MG1655	b4241	treR
1762313_s_at	MG1655	b3209	yhbL
1762314_s_at	MG1655	b1092	fabD
1762315_s_at	MG1655	b0461	ybaJ
1762327_s_at	MG1655	b3406	greB
1762331_s_at	MG1655	b2470	acrD
1762334_s_at	MG1655	b3637	rpmB
1762338_s_at	MG1655	b0147	NA
1762339_s_at	MG1655	b2690	yqaB
1762341_s_at	MG1655	b1658	purR
1762345_s_at	MG1655	b1681	NA
1762360_s_at	MG1655	b1956	NA
1762364_s_at	MG1655	b4374	yjjG
1762367_s_at	MG1655	b3919	tpiA
1762370_s_at	MG1655	b2924	yggB
1762371_s_at	MG1655	b4001	yjaH
1762374_s_at	EDL933	Z5452	rhaT
1762381_s_at	MG1655	b3864	spf
1762383_s_at	MG1655	b1604	NA
1762392_s_at	MG1655	b0116	lpdA
1762395_x_at	CFT073	c2375	NA
1762415_s_at	MG1655	b0049	apaH

1762421_s_at	MG1655	b3061	ttdA
1762423_s_at	MG1655	b3037	ygiB
1762432_s_at	MG1655	b3790	wecD
1762437_s_at	MG1655	b0910	cmk
1762439_s_at	MG1655	b1792	yeaO
1762440_s_at	EDL933	Z3143	NA
1762442_s_at	MG1655	b2314	dedD
1762449_s_at	CFT073	c0385	NA
1762452_s_at	MG1655	b4366	bglJ
1762453_s_at	MG1655	b4171	miaA
1762454_s_at	MG1655	b3318	rplW
1762488_s_at	MG1655	b1639	ydhA
1762494_s_at	MG1655	b1267	yciO
1762497_s_at	MG1655	b2823	ppdC
1762500_s_at	MG1655	b2153	folE
1762501_s_at	MG1655	b3893	fdoH
1762506_s_at	MG1655	b2325	NA
1762512_s_at	MG1655	b0911	rpsA
1762518_s_at	MG1655	b3420	NA
1762520_s_at	MG1655	b3492	yhiN
1762525_s_at	MG1655	b1685	NA
1762530_s_at	MG1655	b1306	pspC
1762531_s_at	MG1655	b2177	yejA
1762534_s_at	MG1655	b1448	NA
1762537_s_at	CFT073	c3902	NA
1762539_s_at	MG1655	b3471	yhhQ
1762543_s_at	MG1655	b0438	clpX
1762547_s_at	MG1655	b3028	mdaB
1762548_s_at	MG1655	b0469	apt
1762551_s_at	MG1655	b4259	holC
1762554_s_at	MG1655	b0156	yadR
1762560_s_at	MG1655	b2414	cysK
1762569_s_at	MG1655	b1875	yecM
1762570_s_at	MG1655	b3014	yqhH
1762572_s_at	MG1655	b3962	udhA
1762576_s_at	MG1655	b1120	cobB
1762577_s_at	MG1655	b3710	yidY
1762595_s_at	MG1655	b0365	tauA
1762596_s_at	SAKAI	ECs1673	NA
1762603_s_at	MG1655	b1324	tpx
1762606_s_at	MG1655	b2198	ccmD
1762610_s_at	CFT073	c0006	NA
1762614_s_at	CFT073	c0813	NA
1762626_s_at	MG1655	b0884	infA
1762627_s_at	MG1655	b2840	ygeA
1762633_s_at	MG1655	b0400	phoR
1762634_s_at	EDL933	Z1317	NA
1762635_s_at	MG1655	b1971	NA
1762640_s_at	MG1655	b3770	ilvE
1762646_s_at	MG1655	b2552	hmpA
1762651_s_at	MG1655	b0474	adk

1762657_s_at	MG1655	b2666	NA
1762664_s_at	MG1655	b3664	yicO
1762674_s_at	MG1655	b0996	torC
1762683_s_at	MG1655	b1333	ydaA
1762688_s_at	CFT073	c0550	NA
1762699_at	MG1655	b4439	micF
1762701_s_at	EDL933	Z5978	NA
1762705_s_at	MG1655	b4196	sgaH
1762706_s_at	MG1655	b1279	yciS
1762707_at	CFT073	c4814	NA
1762710_s_at	MG1655	b0742	ybgF
1762720_s_at	CFT073	c4300	NA
1762726_s_at	MG1655	b1133	NA
1762733_s_at	MG1655	b3508	yhiD
1762735_s_at	MG1655	b3448	yhhA
1762736_s_at	MG1655	b0450	glnK
1762741_s_at	MG1655	b1897	otsB
1762743_s_at	CFT073	c4182	NA
1762748_s_at	MG1655	b3169	nusA
1762751_s_at	MG1655	b0146	sfsA
1762754_s_at	MG1655	b2187	yejL
1762757_s_at	MG1655	b0741	pal
1762761_s_at	MG1655	b3190	yrbA
1762762_s_at	MG1655	b3943	yijE
1762763_s_at	MG1655	b3550	yiaC
1762767_s_at	MG1655	b2990	hybG
1762768_s_at	MG1655	b3297	rpsK
1762772_s_at	EDL933	Z4400	NA
1762774_s_at	MG1655	b0124	gcd
1762777_s_at	MG1655	b2678	proW
1762794_s_at	MG1655	b0458	ylaC
1762810_s_at	MG1655	b1632	NA
1762811_s_at	MG1655	b0101	yacG
1762813_s_at	MG1655	b3781	trxA
1762814_s_at	MG1655	b3713	yieF
1762816_s_at	MG1655	b2154	yeiG
1762819_s_at	CFT073	c4652	pstC
1762821_s_at	MG1655	b0793	ybhS
1762825_s_at	MG1655	b2528	NA
1762830_s_at	MG1655	b2689	NA
1762831_s_at	CFT073	c0481	NA
1762832_s_at	MG1655	b1725	NA
1762836_s_at	MG1655	b3609	secB
1762837_s_at	MG1655	b3164	pnp
1762846_s_at	MG1655	b3984	rplA
1762848_s_at	MG1655	b3100	NA
1762854_s_at	MG1655	b2910	ygfE
1762856_s_at	MG1655	b1670	ydhU
1762857_s_at	MG1655	b2156	lysP
1762860_s_at	CFT073	c1946	yneG
1762864_s_at	MG1655	b3835	NA

1762871_s_at	MG1655	b0712	ybgK
1762872_s_at	MG1655	b3159	yhbV
1762877_s_at	MG1655	b2257	arnT
1762883_s_at	MG1655	b0919	NA
1762884_s_at	MG1655	b1179	ycgL
1762886_s_at	MG1655	b0604	dsbG
1762888_s_at	MG1655	b1285	yciR
1762891_s_at	MG1655	b0451	amtB
1762895_s_at	MG1655	b2216	yojN
1762904_s_at	MG1655	b0864	artP
1762915_s_at	MG1655	b0157	yadS
1762917_s_at	MG1655	b1101	ptsG
1762922_s_at	MG1655	b0152	fhuD
1762933_s_at	MG1655	b3341	rpsG
1762944_s_at	CFT073	c0522	yajI
1762948_s_at	MG1655	b3462	ftsX
1762961_s_at	CFT073	c5293	NA
1762962_s_at	MG1655	b4352	yjiA
1762964_s_at	MG1655	b2400	gltX
1762965_s_at	MG1655	b1955	NA
1762976_s_at	MG1655	b3862	yihG
1762978_s_at	MG1655	b3686	ibpB
1762983_s_at	MG1655	b1070	flgN
1762990_s_at	MG1655	b2423	cysW
1762993_s_at	MG1655	b1851	edd
1762994_s_at	MG1655	b0171	pyrH
1762995_s_at	MG1655	b1128	ycfD
1762998_s_at	CFT073	c1968	NA
1763002_s_at	MG1655	b0964	NA
1763003_s_at	MG1655	b2807	ygdD
1763004_s_at	MG1655	b2496	NA
1763008_s_at	MG1655	b3307	rpsN
1763018_s_at	MG1655	b0028	NA
1763019_s_at	MG1655	b0224	yafK
1763020_s_at	MG1655	b2327	yfcA
1763022_s_at	MG1655	b1663	NA
1763025_s_at	MG1655	b4387	smp
1763027_s_at	MG1655	b0491	ybbM
1763031_s_at	MG1655	b0642	leuS
1763034_s_at	CFT073	c3026	guaA
1763040_s_at	MG1655	b1599	NA
1763043_s_at	MG1655	b2232	ubiG
1763047_s_at	MG1655	b0792	ybhR
1763054_s_at	MG1655	b0839	dacC
1763055_s_at	MG1655	b2313	cvpA
1763057_s_at	MG1655	b1208	NA
1763059_s_at	MG1655	b4453	ldrD
1763066_s_at	MG1655	b1724	NA
1763069_s_at	MG1655	b1818	manY
1763072_s_at	MG1655	b2806	ygdE
1763081_s_at	MG1655	b0830	NA

1763089_s_at	MG1655	b2911	ssrS
1763091_s_at	MG1655	b3366	nirD
1763092_at	MG1655	b4444	rygA
1763093_s_at	CFT073	c0902	NA
1763099_s_at	CFT073	c2740	NA
1763100_s_at	MG1655	b0047	kefC
1763111_s_at	MG1655	b3768	ilvG_2
1763114_s_at	MG1655	b3002	yqhA
1763116_s_at	MG1655	b1072	flgA
1763118_s_at	MG1655	b4160	psd
1763119_s_at	MG1655	b4363	yjjB
1763126_s_at	CFT073	c0746	NA
1763139_s_at	MG1655	b3953	frwD
1763142_s_at	MG1655	b0369	hemB
1763143_s_at	MG1655	b1274	topA
1763145_s_at	MG1655	b3793	wecF
1763146_s_at	MG1655	b1178	ycgK
1763152_s_at	MG1655	b3951	pflD
1763154_s_at	MG1655	b2066	udk
1763160_s_at	MG1655	b3202	rpoN
1763164_s_at	MG1655	b2431	NA
1763167_s_at	MG1655	b3762	yifA
1763169_s_at	MG1655	b4383	deoB
1763170_s_at	CFT073	c2257	NA
1763185_s_at	CFT073	c5221	NA
1763188_s_at	MG1655	b1277	ribA
1763190_s_at	MG1655	b3025	ygiX
1763194_s_at	MG1655	b0477	gsk
1763199_s_at	CFT073	c4882	NA
1763206_s_at	MG1655	b4391	yjjK
1763211_s_at	CFT073	c0841	modB
1763212_s_at	MG1655	b3127	garP
1763213_s_at	MG1655	b2347	yfdC
1763216_s_at	MG1655	b4434	IS092
1763217_s_at	CFT073	c0827	NA
1763221_s_at	MG1655	b0867	NA
1763225_s_at	SAKAI	ECs5185	NA
1763230_s_at	CFT073	c2839	NA
1763233_s_at	MG1655	b0195	yaeB
1763234_s_at	MG1655	b2596	NA
1763236_s_at	MG1655	b1013	ycdC
1763242_s_at	MG1655	b0951	pqiB
1763243_s_at	MG1655	b0685	ybfE
1763249_s_at	MG1655	b4057	yjbR
1763257_s_at	MG1655	b3507	yhiF
1763259_s_at	MG1655	b0406	tgt
1763260_s_at	CFT073	c0754	NA
1763264_s_at	MG1655	b2410	yfeH
1763265_s_at	MG1655	b0215	dnaQ
1763267_s_at	MG1655	b1790	yeaM
1763269_x_at	CFT073	c1013	NA

1763273_s_at	MG1655	b3908	sodA
1763275_s_at	MG1655	b3752	rbsK
1763282_s_at	EDL933	Z4148	NA
1763284_s_at	MG1655	b3068	ygjF
1763290_s_at	SAKAI	ECs3895	NA
1763298_s_at	MG1655	b2951	yggS
1763307_s_at	MG1655	b1630	NA
1763308_s_at	MG1655	b3404	envZ
1763312_at	MG1655	b4408	csrB
1763317_s_at	MG1655	b1292	sapC
1763323_s_at	MG1655	b3109	yhaN
1763324_s_at	MG1655	b1235	hnr
1763331_s_at	CFT073	c0062	NA
1763332_s_at	MG1655	b3393	yrfB
1763333_s_at	CFT073	c4476	spoU
1763334_s_at	EDL933	Z2664	NA
1763336_s_at	MG1655	b0180	fabZ
1763344_s_at	MG1655	b3465	yhhF
1763346_s_at	MG1655	b2959	yggL
1763350_s_at	MG1655	b1221	narL
1763357_s_at	MG1655	b0014	dnaK
1763361_s_at	MG1655	b4222	ytfP
1763364_s_at	MG1655	b0896	dmsC
1763374_s_at	CFT073	c1053	NA
1763386_s_at	CFT073	c2108	NA
1763389_s_at	MG1655	b0174	NA
1763391_s_at	MG1655	b0476	NA
1763396_s_at	CFT073	c0820	NA
1763400_s_at	MG1655	b3938	metJ
1763403_s_at	MG1655	b3750	rbsC
1763404_s_at	MG1655	b1110	ycfJ
1763406_s_at	MG1655	b1524	yneH
1763416_s_at	MG1655	b1108	ycfP
1763422_s_at	CFT073	c3139	NA
1763430_s_at	MG1655	b3636	rpmG
1763438_s_at	MG1655	b3064	ygjD
1763444_s_at	MG1655	b1117	ycfV
1763446_s_at	MG1655	b4140	NA
1763453_s_at	MG1655	b3867	hemN
1763464_s_at	MG1655	b2203	napB
1763466_s_at	MG1655	b2743	pcm
1763473_s_at	MG1655	b2587	kgtP
1763476_s_at	SAKAI	ECs5165	NA
1763486_s_at	MG1655	b1088	yceD
1763488_s_at	MG1655	b1636	pdxY
1763490_s_at	MG1655	b1922	fliA
1763494_s_at	SAKAI	ECs3674	NA
1763499_s_at	MG1655	b1225	narH
1763500_s_at	SAKAI	ECs5537	NA
1763503_s_at	MG1655	b1620	malI
1763508_s_at	MG1655	b2159	nfo

1763510_s_at	MG1655	b1740	nadE
1763516_s_at	MG1655	b1060	yceP
1763518_s_at	CFT073	c4889	NA
1763530_s_at	CFT073	c1112	NA
1763531_s_at	MG1655	b2085	NA
1763534_at	CFT073	c5055	NA
1763535_s_at	MG1655	b0479	fsr
1763536_s_at	MG1655	b3742	mioC
1763537_s_at	MG1655	b0923	mukE
1763538_s_at	MG1655	b3389	aroB
1763542_s_at	MG1655	b1273	yciN
1763551_s_at	MG1655	b1852	zwf
1763569_s_at	MG1655	b3282	yrdC
1763579_s_at	MG1655	b2896	NA
1763580_s_at	MG1655	b3648	gmk
1763581_s_at	MG1655	b0386	proC
1763582_s_at	MG1655	b2065	dcd
1763585_s_at	EDL933	Z0281	yafJ
1763589_s_at	MG1655	b1035	ycdY
1763593_s_at	MG1655	b3354	yheU
1763600_s_at	CFT073	c1108	NA
1763603_s_at	MG1655	b1234	ychK
1763608_s_at	MG1655	b2128	yehW
1763610_s_at	MG1655	b1105	ycfM
1763611_s_at	EDL933	Z4822	ugpB
1763619_s_at	MG1655	b2007	yeeX
1763622_s_at	MG1655	b3674	yidF
1763626_s_at	MG1655	b1266	yciV
1763638_s_at	MG1655	b1716	rplT
1763642_s_at	MG1655	b3528	dctA
1763649_s_at	CFT073	c1109	NA
1763652_s_at	MG1655	b2925	NA
1763653_s_at	MG1655	b2837	galR
1763657_s_at	CFT073	c3928	NA
1763663_s_at	MG1655	b0072	leuC
1763668_s_at	MG1655	b3229	sspA
1763669_s_at	CFT073	c3486	NA
1763679_at	MG1655	b4438	ryeE
1763680_s_at	MG1655	b0079	fruL
1763681_s_at	MG1655	b0449	mdlB
1763683_s_at	CFT073	c2061	NA
1763695_s_at	MG1655	b3906	rhaR
1763697_s_at	MG1655	b0081	yabB
1763700_s_at	MG1655	b1654	ydhD
1763705_s_at	CFT073	c3821	rpoD
1763709_s_at	MG1655	b0437	clpP
1763712_s_at	MG1655	b2480	bcp
1763716_s_at	MG1655	b1809	NA
1763717_s_at	MG1655	b0219	yafV
1763718_s_at	MG1655	b1113	ycfS
1763719_s_at	CFT073	c3355	NA

1763723_s_at	MG1655	b0154	hemL
1763724_s_at	MG1655	b3733	atpG
1763727_s_at	CFT073	c2967	yfeZ
1763729_s_at	MG1655	b3857	mobA
1763732_s_at	MG1655	b4118	melR
1763733_s_at	MG1655	b3748	rbsD
1763744_s_at	MG1655	b3165	rpsO
1763749_s_at	MG1655	b1596	ynfM
1763752_s_at	MG1655	b4400	creD
1763768_s_at	EDL933	Z4059	cysN
1763777_s_at	MG1655	b0590	fepD
1763783_s_at	MG1655	b4043	lexA
1763790_s_at	MG1655	b2569	lepA
1763796_s_at	MG1655	b2785	NA
1763803_at	CFT073	c4626	NA
1763806_s_at	CFT073	c1010	NA
1763816_s_at	MG1655	b3639	dfp
1763822_s_at	EDL933	Z5042	yibD
1763825_s_at	CFT073	c1642	NA
1763829_s_at	MG1655	b0240	crl
1763830_s_at	MG1655	b1729	NA
1763831_s_at	MG1655	b0092	ddlB
1763836_s_at	MG1655	b4232	fbp
1763838_s_at	MG1655	b3461	rpoH
1763845_s_at	MG1655	b1284	NA
1763861_s_at	MG1655	b0924	mukB
1763865_s_at	MG1655	b3920	yiiQ
1763870_s_at	MG1655	b0456	ybaA
1763871_s_at	MG1655	b0094	ftsA
1763873_s_at	MG1655	b3363	ppiA
1763881_s_at	MG1655	b0733	cydA
1763897_s_at	MG1655	b2286	nuoC
1763906_s_at	MG1655	b3436	gntU_1
1763908_s_at	MG1655	b0187	yaeR
1763921_s_at	MG1655	b2015	yeeY
1763922_s_at	MG1655	b3170	yhbC
1763924_s_at	CFT073	c4074	NA
1763926_s_at	MG1655	b1669	NA
1763927_s_at	CFT073	c3354	NA
1763931_s_at	MG1655	b2237	inaA
1763938_s_at	MG1655	b0785	moaE
1763947_s_at	MG1655	b1061	dinI
1763948_s_at	MG1655	b0779	uvrB
1763957_s_at	MG1655	b0082	yabC
1763970_s_at	MG1655	b2808	gcvA
1763971_at	CFT073	c4934	NA
1763973_s_at	MG1655	b1214	ychA
1763981_s_at	MG1655	b4015	aceA
1763985_s_at	MG1655	b0455	ffs
1763987_s_at	MG1655	b2804	fucU
1763995_s_at	MG1655	b1475	fdnH

1763997_s_at	MG1655	b3725	pstB
1764003_s_at	MG1655	b0098	secA
1764004_s_at	MG1655	b2952	yggT
1764006_s_at	MG1655	b3346	yheO
1764019_s_at	MG1655	b3012	NA
1764020_s_at	CFT073	c1478	NA
1764033_s_at	MG1655	b1925	fliS
1764050_s_at	MG1655	b0396	araJ
1764061_s_at	CFT073	c0103	murE
1764066_s_at	MG1655	b4029	yjbH
1764073_s_at	MG1655	b3239	yhcO
1764076_s_at	MG1655	b0213	yafS
1764080_s_at	MG1655	b2673	nrdH
1764083_s_at	MG1655	b4000	hupA
1764086_s_at	MG1655	b3200	yhbN
1764093_s_at	CFT073	c3478	NA
1764098_s_at	MG1655	b3154	yhbP
1764100_s_at	MG1655	b4381	deoC
1764101_s_at	MG1655	b4006	purH
1764102_s_at	MG1655	b1055	yceA
1764112_s_at	MG1655	b2293	yfbT
1764113_s_at	MG1655	b3700	recF
1764120_s_at	MG1655	b2321	NA
1764134_s_at	CFT073	c4007	NA
1764140_s_at	MG1655	b0126	yadF
1764141_s_at	MG1655	b3250	mreC
1764144_s_at	CFT073	c4808	NA
1764146_s_at	MG1655	b1973	NA
1764149_s_at	EDL933	Z0642	ybbK
1764156_s_at	MG1655	b1813	yeaB
1764162_s_at	EDL933	Z0202	NA
1764164_s_at	MG1655	b1039	csgE
1764167_s_at	MG1655	b1215	kdsA
1764175_s_at	MG1655	b2531	iscR
1764176_s_at	MG1655	b0688	pgm
1764177_s_at	MG1655	b3910	yiiM
1764178_s_at	MG1655	b3256	accC
1764179_s_at	CFT073	c3379	NA
1764183_s_at	MG1655	b4394	yjjX
1764186_s_at	MG1655	b3085	ygjP
1764190_s_at	MG1655	b0522	purK
1764198_s_at	MG1655	b3197	yrbH
1764199_s_at	SAKAI	ECs5391	NA
1764200_s_at	MG1655	b0874	ybjE
1764209_s_at	MG1655	b2467	yffH
1764211_s_at	MG1655	b2382	NA
1764215_s_at	CFT073	c4431	NA
1764217_s_at	CFT073	c1074	NA
1764226_s_at	MG1655	b3611	yibN
1764229_s_at	MG1655	b2663	gabP
1764230_s_at	MG1655	b1233	ychJ

1764234_s_at	MG1655	b0401	brnQ
1764239_s_at	MG1655	b0029	NA
1764240_s_at	MG1655	b2464	talA
1764243_s_at	CFT073	c0840	modA
1764251_s_at	MG1655	b0110	ampD
1764252_at	SAKAI	ECs0701	NA
1764253_s_at	MG1655	b1779	gapA
1764254_s_at	MG1655	b2601	aroF
1764258_s_at	CFT073	c2687	yeiB
1764259_s_at	MG1655	b4041	plsB
1764262_s_at	MG1655	b0891	lolA
1764272_s_at	MG1655	b0104	guaC
1764274_s_at	MG1655	b1044	NA
1764281_s_at	MG1655	b0416	nusB
1764292_s_at	MG1655	b1063	yceB
1764293_s_at	MG1655	b3098	yqjD
1764294_s_at	MG1655	b0878	NA
1764295_s_at	MG1655	b0880	cspD
1764303_s_at	MG1655	b3199	yrbK
1764305_s_at	MG1655	b2392	NA
1764306_s_at	MG1655	b3704	rnpA
1764311_s_at	MG1655	b3988	rpoC
1764316_s_at	CFT073	c4228	yhhW
1764321_s_at	MG1655	b0796	ybiH
1764323_s_at	MG1655	b1223	narK
1764325_s_at	MG1655	b1682	NA
1764328_s_at	MG1655	b1907	tyrP
1764330_s_at	MG1655	b1976	NA
1764331_s_at	MG1655	b2167	fruA
1764337_s_at	CFT073	c0222	NA
1764349_s_at	MG1655	b4056	yjbQ
1764359_s_at	MG1655	b3930	menA
1764360_s_at	CFT073	c0510	NA
1764362_s_at	MG1655	b2474	ypfI
1764370_s_at	MG1655	b2579	yfiD
1764381_s_at	MG1655	b3736	atpF
1764390_s_at	CFT073	c4090	NA
1764395_s_at	MG1655	b2175	spr
1764399_s_at	MG1655	b0006	yaaA
1764402_s_at	MG1655	b3952	pflC
1764403_s_at	MG1655	b3993	thiE
1764405_s_at	MG1655	b0682	ybfN
1764409_s_at	MG1655	b4226	ppa
1764411_s_at	MG1655	b2178	yejB
1764413_s_at	MG1655	b1856	yebA
1764425_s_at	MG1655	b3591	selA
1764432_s_at	MG1655	b0841	ybjG
1764433_s_at	MG1655	b2570	rseC
1764435_s_at	MG1655	b3038	ygiC
1764436_s_at	MG1655	b3245	yhdP
1764438_s_at	MG1655	b1900	araG

1764439_s_at	MG1655	b0870	NA
1764457_s_at	MG1655	b3189	murA
1764458_s_at	MG1655	b2794	yqcD
1764467_s_at	MG1655	b3228	sspB
1764468_s_at	MG1655	b0096	lpxC
1764470_s_at	MG1655	b0389	yaiA
1764471_s_at	MG1655	b0622	crcA
1764473_s_at	MG1655	b1250	kch
1764474_at	CFT073	c4999	NA
1764478_s_at	CFT073	c4294	NA
1764480_s_at	MG1655	b3390	aroK
1764483_s_at	MG1655	b1743	spy
1764487_s_at	MG1655	b4255	yjgD
1764489_s_at	CFT073	c1062	NA
1764493_s_at	MG1655	b2607	trmD
1764497_s_at	MG1655	b2529	NA
1764505_s_at	MG1655	b4108	phnA
1764513_s_at	MG1655	b3024	ygiW
1764514_s_at	MG1655	b1692	ydiB
1764520_s_at	MG1655	b2151	galS
1764521_s_at	MG1655	b3735	atpH
1764522_s_at	EDL933	Z5717	yjdE
1764525_s_at	MG1655	b3066	dnaG
1764535_s_at	MG1655	b2953	yggU
1764537_s_at	MG1655	b2499	purM
1764557_s_at	MG1655	b1183	umuD
1764559_s_at	MG1655	b0058	NA
1764563_s_at	CFT073	c4663	NA
1764571_s_at	MG1655	b1054	htrB
1764572_s_at	MG1655	b3016	ygiR
1764573_s_at	MG1655	b2157	yeiE
1764575_s_at	CFT073	c4059	NA
1764582_s_at	MG1655	b1288	fabI
1764586_s_at	MG1655	b4245	pyrB
1764589_s_at	MG1655	b3820	yigI
1764591_s_at	MG1655	b0961	yccF
1764593_s_at	MG1655	b0214	rnhA
1764594_s_at	MG1655	b0815	ybiP
1764600_s_at	CFT073	c2535	NA
1764607_s_at	MG1655	b2239	glpQ
1764625_s_at	MG1655	b2795	ygdH
1764630_s_at	MG1655	b4060	yjcB
1764631_s_at	MG1655	b3787	wecC
1764632_s_at	MG1655	b1709	btuD
1764637_s_at	MG1655	b3865	yihA
1764659_s_at	MG1655	b2234	nrdA
1764660_s_at	CFT073	c3827	NA
1764663_s_at	CFT073	c2949	NA
1764667_s_at	MG1655	b3668	uhpB
1764668_s_at	MG1655	b0007	yaaJ
1764678_at	MG1655	b4456	ryiA

1764679_s_at	CFT073	c5009	ubiC
1764680_s_at	MG1655	b3352	yheS
1764686_s_at	MG1655	b2594	NA
1764696_s_at	MG1655	b2821	ptr
1764702_s_at	MG1655	b3260	yhdG
1764704_s_at	MG1655	b2513	NA
1764713_s_at	MG1655	b3555	yiaG
1764714_s_at	MG1655	b1710	btuE
1764724_s_at	MG1655	b0093	ftsQ
1764725_s_at	MG1655	b0866	NA
1764726_s_at	MG1655	b3478	nikC
1764731_s_at	MG1655	b2475	NA
1764737_s_at	MG1655	b1830	prc
1764746_s_at	EDL933	Z0828	NA
1764761_s_at	MG1655	b4455	hokA
1764762_s_at	MG1655	b1819	manZ
1764771_s_at	MG1655	b0926	ycbK
1764775_s_at	MG1655	b2939	yqgB
1764776_s_at	MG1655	b3146	yraL
1764786_s_at	MG1655	b1633	nth
1764788_s_at	MG1655	b3493	pitA
1764794_s_at	MG1655	b3301	rplO
1764795_s_at	MG1655	b2893	dsbC
1764799_s_at	MG1655	b4364	yjjP
1764804_s_at	MG1655	b3728	pstS
1764811_s_at	MG1655	b1190	dadX
1764813_s_at	MG1655	b3403	pckA
1764814_s_at	MG1655	b0121	speE
1764819_s_at	CFT073	c4909	NA
1764822_s_at	MG1655	b3172	argG
1764826_s_at	MG1655	b0627	NA
1764833_s_at	MG1655	b0160	dgt
1764837_s_at	MG1655	b1672	NA
1764839_s_at	MG1655	b2196	ccmF
1764871_s_at	MG1655	b2592	clpB
1764873_s_at	MG1655	b3399	yrfG
1764874_s_at	MG1655	b1664	NA
1764881_s_at	MG1655	b0625	ybeH
1764892_s_at	MG1655	b3196	yrbG
1764893_s_at	MG1655	b2343	NA
1764894_s_at	MG1655	b4142	NA
1764900_s_at	MG1655	b2188	yejM
1764901_s_at	MG1655	b2417	crr
1764904_s_at	MG1655	b0958	sulA
1764917_s_at	CFT073	c2686	NA
1764921_s_at	MG1655	b2498	upp
1764922_s_at	EDL933	Z2533	NA
1764923_s_at	MG1655	b1952	dsrB
1764937_s_at	MG1655	b0998	torD
1764952_s_at	MG1655	b3108	yhaM
1764967_s_at	CFT073	c2742	NA

1764969_s_at	MG1655	b1243	oppA
1764974_s_at	MG1655	b2922	yggE
1764976_s_at	MG1655	b3468	yhhN
1764983_s_at	MG1655	b3914	NA
1765002_s_at	MG1655	b4190	yjfP
1765014_s_at	MG1655	b0066	yabJ
1765016_s_at	MG1655	b0822	NA
1765021_s_at	CFT073	c2806	NA
1765022_s_at	MG1655	b4040	ubiA
1765030_s_at	CFT073	c4998	NA
1765036_s_at	MG1655	b0966	yccV
1765044_s_at	MG1655	b3649	rpoZ
1765046_s_at	MG1655	b3525	yhjH
1765047_s_at	MG1655	b1855	msbB
1765050_s_at	MG1655	b3290	trkA
1765056_s_at	SAKAI	ECs5363	NA
1765060_s_at	MG1655	b3643	rph
1765061_s_at	EDL933	Z4368	yqhG
1765066_s_at	MG1655	b3933	ftsN
1765067_s_at	MG1655	b3314	rpsC
1765071_s_at	MG1655	b0683	fur
1765076_s_at	MG1655	b1869	yecN
1765087_s_at	MG1655	b0577	ybdG
1765089_s_at	MG1655	b3654	yicE
1765090_s_at	EDL933	Z3849	NA
1765091_at	CFT073	c4867	pfkA
1765092_s_at	MG1655	b2748	NA
1765096_s_at	MG1655	b4409	blr
1765099_s_at	MG1655	b0851	NA
1765108_s_at	MG1655	b1272	sohB
1765110_s_at	MG1655	b4153	frdB
1765114_s_at	MG1655	b0892	ycaJ
1765116_s_at	MG1655	b0955	NA
1765133_s_at	MG1655	b0989	cspH
1765136_s_at	MG1655	b3180	yhbY
1765151_s_at	MG1655	b3303	rpsE
1765153_s_at	MG1655	b4119	melA
1765166_s_at	CFT073	c2371	NA
1765167_s_at	MG1655	b3928	yiiU
1765168_s_at	MG1655	b2303	folX
1765170_s_at	MG1655	b3397	yrfE
1765171_s_at	MG1655	b4050	yjbO
1765175_s_at	MG1655	b2609	rpsP
1765179_s_at	MG1655	b0954	fabA
1765183_s_at	MG1655	b0807	ybiN
1765186_s_at	MG1655	b0781	moaA
1765192_s_at	MG1655	b2210	NA
1765194_s_at	MG1655	b1822	NA
1765195_s_at	MG1655	b1789	yeaL
1765201_s_at	CFT073	c3016	NA
1765202_s_at	MG1655	b0643	ybeL

1765207_s_at	MG1655	b3296	rpsD
1765214_s_at	MG1655	b4414	t44
1765215_s_at	MG1655	b3254	NA
1765216_s_at	MG1655	b3816	corA
1765225_s_at	MG1655	b2829	ptsP
1765229_s_at	MG1655	b0067	yabK
1765230_s_at	MG1655	b2903	gcvP
1765232_s_at	MG1655	b0934	NA
1765233_s_at	MG1655	b0596	entA
1765234_s_at	MG1655	b2296	ackA
1765248_s_at	MG1655	b0995	torR
1765249_s_at	MG1655	b0388	aroL
1765261_s_at	MG1655	b0838	yliJ
1765262_s_at	MG1655	b0050	apaG
1765263_s_at	MG1655	b3157	yhbT
1765266_s_at	MG1655	b2894	xerD
1765269_s_at	MG1655	b2559	tadA
1765272_s_at	MG1655	b0172	frr
1765273_s_at	MG1655	b0183	rnhB
1765277_s_at	MG1655	b3850	hemG
1765284_s_at	MG1655	b2597	yfiA
1765285_s_at	MG1655	b0504	ybbS
1765297_s_at	MG1655	b1807	yeaZ
1765299_s_at	CFT073	c2248	NA
1765308_s_at	EDL933	Z2519	NA
1765309_s_at	MG1655	b0680	glnS
1765313_s_at	MG1655	b2905	gcvT
1765327_s_at	MG1655	b1332	ynaJ
1765332_s_at	MG1655	b3094	exuR
1765341_s_at	MG1655	b3097	yqjC
1765354_s_at	MG1655	b0912	himD
1765355_at	MG1655	b0816	NA
1765369_s_at	MG1655	b1826	NA
1765370_s_at	MG1655	b1728	NA
1765371_s_at	MG1655	b1739	osmE
1765372_s_at	CFT073	c1751	yciH
1765380_s_at	MG1655	b3608	gpsA
1765385_at	CFT073	c4903	NA
1765388_s_at	MG1655	b3934	cytR
1765389_s_at	MG1655	b2824	ygdB
1765390_s_at	MG1655	b1213	NA
1765395_s_at	MG1655	b1132	ycfC
1765397_s_at	MG1655	b3466	yhhL
1765413_s_at	MG1655	b1034	ycdX
1765417_s_at	MG1655	b3119	tdcR
1765419_s_at	SAKAI	ECs2526	NA
1765427_s_at	MG1655	b2904	gcvH
1765431_s_at	MG1655	b3067	rpoD
1765433_at	MG1655	b4431	rprA
1765435_s_at	MG1655	b0653	gltK
1765438_s_at	CFT073	c3470	NA

1765440_s_at	MG1655	b0433	ampG
1765450_s_at	CFT073	c0192	NA
1765451_s_at	MG1655	b3191	yrbB
1765452_s_at	MG1655	b3961	oxyR
1765453_s_at	MG1655	b0972	hyaA
1765454_s_at	MG1655	b3783	rho
1765458_s_at	MG1655	b0804	ybiX
1765459_s_at	MG1655	b2836	aas
1765464_s_at	MG1655	b1095	fabF
1765474_s_at	MG1655	b0956	ycbG
1765498_s_at	CFT073	c0823	NA
1765505_s_at	CFT073	c5246	NA
1765508_s_at	MG1655	b0013	yaaI
1765513_s_at	MG1655	b1247	oppF
1765516_s_at	MG1655	b4173	hflX
1765518_s_at	MG1655	b0595	entB
1765520_s_at	MG1655	b0994	torT
1765529_s_at	MG1655	b0208	yafC
1765533_s_at	MG1655	b3701	dnaN
1765538_s_at	MG1655	b1717	rpmI
1765539_s_at	MG1655	b2926	pgk
1765540_s_at	CFT073	c3818	NA
1765541_s_at	MG1655	b0387	yaiI
1765545_s_at	MG1655	b2278	nuoL
1765549_s_at	MG1655	b3019	parC
1765550_s_at	MG1655	b1185	dsbB
1765553_s_at	MG1655	b3054	ygiF
1765561_s_at	MG1655	b4203	rplI
1765562_s_at	CFT073	c1673	NA
1765576_s_at	CFT073	c5229	NA
1765578_s_at	MG1655	b3039	ygiD
1765585_s_at	MG1655	b4051	qor
1765588_s_at	MG1655	b0734	cydB
1765591_s_at	MG1655	b0418	pgpA
1765594_s_at	MG1655	b3732	atpD
1765597_s_at	MG1655	b1841	NA
1765609_s_at	MG1655	b3175	secG
1765610_s_at	MG1655	b1188	ycgB
1765622_s_at	MG1655	b1657	NA
1765631_s_at	MG1655	b3775	ppiC
1765637_s_at	MG1655	b0580	ybdJ
1765652_s_at	MG1655	b0440	hupB
1765659_s_at	MG1655	b2780	pyrG
1765667_s_at	MG1655	b2412	zipA
1765669_s_at	MG1655	b2386	NA
1765680_s_at	MG1655	b4361	dnaC
1765689_s_at	MG1655	b4375	prfC
1765694_s_at	MG1655	b2473	ypfH
1765695_s_at	MG1655	b1847	yebF
1765696_s_at	MG1655	b2140	yohI
1765697_s_at	MG1655	b3320	rplC

1765704_s_at	MG1655	b3916	pfkA
1765707_s_at	MG1655	b2841	araE
1765712_s_at	MG1655	b3385	gph
1765717_s_at	MG1655	b3958	argC
1765728_s_at	MG1655	b0694	kdpE
1765732_s_at	MG1655	b2208	napF
1765736_s_at	MG1655	b1204	pth
1765738_s_at	CFT073	c4780	NA
1765749_s_at	MG1655	b3302	rpmD
1765754_s_at	MG1655	b1843	NA
1765755_s_at	MG1655	b3992	thiF
1765757_s_at	MG1655	b2799	fucO
1765760_s_at	MG1655	b0404	yajB
1765761_s_at	MG1655	b2281	nuoI
1765765_s_at	EDL933	Z0726	NA
1765766_s_at	MG1655	b2472	dapE
1765772_s_at	MG1655	b3559	glyS
1765773_s_at	MG1655	b1245	oppC
1765776_s_at	MG1655	b3057	bacA
1765777_s_at	MG1655	b0714	nei
1765783_s_at	MG1655	b1246	oppD
1765789_s_at	MG1655	b2961	mutY
1765795_s_at	CFT073	c0686	NA
1765796_s_at	MG1655	b1307	pspD
1765798_s_at	MG1655	b1236	galU
1765806_s_at	MG1655	b2262	menB
1765811_s_at	MG1655	b3467	yhhM
1765812_s_at	MG1655	b1930	yedF
1765821_s_at	CFT073	c0474	NA
1765834_s_at	MG1655	b0887	cydD
1765835_s_at	MG1655	b1275	cysB
1765836_s_at	MG1655	b0109	nadC
1765838_s_at	MG1655	b3408	feoA
1765839_s_at	MG1655	b1090	plsX
1765842_s_at	MG1655	b3425	glpE
1765846_s_at	MG1655	b3237	argR
1765847_s_at	MG1655	b2668	ygaP
1765850_s_at	MG1655	b1602	pntB
1765853_s_at	MG1655	b1118	ycfW
1765855_s_at	MG1655	b0242	proB
1765864_s_at	MG1655	b0125	hpt
1765885_s_at	MG1655	b0159	pfs
1765886_s_at	MG1655	b0915	NA
1765887_s_at	MG1655	b0856	potH
1765892_s_at	MG1655	b0713	ybgL
1765899_s_at	CFT073	c1031	dmsA
1765900_s_at	MG1655	b2940	yqgC
1765901_s_at	MG1655	b4172	hfq
1765903_s_at	MG1655	b1954	dsrA
1765920_s_at	MG1655	b2381	NA
1765938_s_at	MG1655	b1303	pspF

1765939_s_at	MG1655	b3179	NA
1765940_s_at	MG1655	b1671	NA
1765941_s_at	MG1655	b1111	ycfQ
1765942_s_at	MG1655	b0003	thrB
1765945_s_at	MG1655	b3981	secE
1765949_s_at	MG1655	b1857	NA
1765962_s_at	MG1655	b1059	solA
1765965_s_at	MG1655	b3032	NA
1765967_s_at	CFT073	c4938	NA
1765968_s_at	MG1655	b2185	rplY
1765974_s_at	MG1655	b3614	yibQ
1765981_s_at	CFT073	c4115	NA
1765982_s_at	MG1655	b0782	moaB
1765984_s_at	MG1655	b2791	yqcB
1765986_s_at	MG1655	b3294	rplQ
1765995_s_at	MG1655	b1629	NA
1765996_s_at	EDL933	Z4757	NA
1766006_s_at	MG1655	b0529	folD
1766008_s_at	MG1655	b3041	ribB
1766014_s_at	EDL933	Z0039	NA
1766025_s_at	CFT073	c3971	yhcC
1766034_s_at	CFT073	c4194	malP
1766049_s_at	MG1655	b0631	ybeD
1766050_s_at	MG1655	b2793	syd
1766061_s_at	MG1655	b2279	nuoK
1766069_s_at	MG1655	b3162	deaD
1766073_s_at	MG1655	b3915	yiiP
1766075_s_at	MG1655	b0849	grxA
1766088_s_at	MG1655	b3457	livH
1766090_s_at	MG1655	b2835	ygeD
1766093_s_at	MG1655	b0486	ybaT
1766098_s_at	MG1655	b3726	pstA
1766104_s_at	MG1655	b3475	NA
1766107_s_at	MG1655	b3744	asnA
1766109_s_at	MG1655	b3805	hemC
1766117_s_at	MG1655	b1764	selD
1766125_s_at	CFT073	c0547	NA
1766127_s_at	MG1655	b1972	NA
1766130_s_at	MG1655	b1842	holE
1766141_s_at	CFT073	c2266	NA
1766145_s_at	CFT073	c2837	NA
1766153_s_at	MG1655	b0222	NA
1766161_s_at	MG1655	b3166	truB
1766162_s_at	MG1655	b2244	yfaD
1766163_s_at	MG1655	b2916	iciA
1766174_s_at	MG1655	b0083	ftsL
1766175_s_at	MG1655	b2568	lepB
1766177_s_at	MG1655	b2415	ptsH
1766180_s_at	MG1655	b0811	glnH
1766187_s_at	MG1655	b2809	NA
1766189_s_at	MG1655	b0633	rlpA

1766190_s_at	MG1655	b3430	glgC
1766204_s_at	MG1655	b3996	NA
1766206_at	MG1655	b4450	ryhA
1766211_s_at	MG1655	b3252	yhdA
1766213_s_at	MG1655	b2469	narQ
1766215_s_at	MG1655	b1100	ycfH
1766217_s_at	MG1655	b3388	damX
1766229_s_at	CFT073	c0120	NA
1766234_at	CFT073	c5464	NA
1766235_s_at	MG1655	b1652	rnt
1766238_s_at	MG1655	b3270	yhdY
1766240_s_at	EDL933	Z5336	NA
1766242_s_at	MG1655	b3226	NA
1766243_s_at	MG1655	b1638	pdxH
1766244_s_at	MG1655	b3635	mutM
1766247_s_at	MG1655	b2524	yfhJ
1766250_s_at	MG1655	b1591	NA
1766252_s_at	MG1655	b0828	ybiK
1766253_s_at	MG1655	b3782	rhoL
1766256_s_at	MG1655	b3423	glpR
1766262_s_at	MG1655	b0739	tolA
1766263_s_at	MG1655	b2941	yqgD
1766267_s_at	CFT073	c0039	NA
1766268_s_at	MG1655	b2989	NA
1766275_s_at	MG1655	b0442	ybaV
1766276_s_at	MG1655	b1129	phoQ
1766277_s_at	MG1655	b2745	ygbO
1766280_s_at	MG1655	b1127	pepT
1766282_s_at	MG1655	b2957	ansB
1766284_s_at	CFT073	c0552	NA
1766288_s_at	EDL933	Z5326	dapF
1766291_s_at	MG1655	b1592	NA
1766292_s_at	MG1655	b1661	cfa
1766295_s_at	CFT073	c2891	NA
1766300_s_at	MG1655	b2290	NA
1766302_s_at	MG1655	b0846	NA
1766306_s_at	MG1655	b0493	ybbO
1766310_s_at	MG1655	b0425	apbA
1766314_s_at	MG1655	b3029	ygiN
1766316_s_at	MG1655	b0011	NA
1766320_s_at	MG1655	b2995	hybB
1766322_at	CFT073	c1111	NA
1766325_s_at	MG1655	b3001	NA
1766326_s_at	MG1655	b3849	trkH
1766332_s_at	MG1655	b3070	yqjH
1766334_s_at	MG1655	b2143	cdd
1766335_s_at	MG1655	b0488	ybbJ
1766336_s_at	MG1655	b0071	leuD
1766338_s_at	MG1655	b3840	NA
1766346_s_at	MG1655	b4258	valS
1766349_s_at	MG1655	b2600	tyrA

1766363_s_at	MG1655	b3184	yhbE
1766365_s_at	MG1655	b4397	creA
1766367_s_at	MG1655	b0710	ybgI
1766374_s_at	MG1655	b0417	thiL
1766378_s_at	CFT073	c2757	NA
1766381_s_at	MG1655	b3909	kdgT
1766387_s_at	MG1655	b1048	mdoG
1766390_s_at	MG1655	b1036	ycdZ
1766397_s_at	EDL933	Z5401	NA
1766400_s_at	MG1655	b3305	rplF
1766402_s_at	MG1655	b4191	yjfQ
1766403_s_at	MG1655	b3974	coaA
1766404_s_at	MG1655	b2553	glnB
1766408_s_at	MG1655	b1927	amyA
1766411_s_at	MG1655	b0095	ftsZ
1766413_s_at	MG1655	b3223	NA
1766415_s_at	CFT073	c1776	NA
1766423_s_at	MG1655	b3641	ttk
1766425_s_at	MG1655	b4201	priB
1766437_s_at	MG1655	b1278	pgpB
1766441_s_at	MG1655	b3185	rpmA
1766450_s_at	MG1655	b1861	ruvA
1766461_s_at	MG1655	b1293	sapB
1766462_s_at	MG1655	b0783	moaC
1766463_s_at	MG1655	b4136	dsbD
1766472_s_at	MG1655	b1249	cls
1766475_s_at	MG1655	b2683	ygaH
1766478_s_at	MG1655	b0212	gloB
1766480_s_at	MG1655	b2377	NA
1766481_s_at	MG1655	b2684	emrR
1766488_s_at	MG1655	b2564	pdxJ
1766489_s_at	MG1655	b0699	ybfA
1766490_s_at	MG1655	b0881	yljA
1766494_s_at	MG1655	b3553	NA
1766499_s_at	MG1655	b1714	pheS
1766506_s_at	CFT073	c4687	yifB
1766511_x_at	CFT073	c5497	pheL
1766513_s_at	MG1655	b0492	ybbN
1766514_s_at	MG1655	b0113	pdhR
1766523_s_at	MG1655	b3344	yheM
1766531_s_at	CFT073	c0695	ahpF
1766535_s_at	MG1655	b2125	yehT
1766543_s_at	MG1655	b0064	araC
1766544_s_at	MG1655	b0457	ylaB
1766547_s_at	MG1655	b2562	yfhL
1766551_s_at	MG1655	b0468	ybaN
1766553_s_at	MG1655	b3785	wzzE
1766558_s_at	MG1655	b0177	yaeT
1766562_s_at	MG1655	b2812	ygdL
1766564_s_at	MG1655	b3959	argB
1766568_s_at	MG1655	b1094	acpP

1766569_s_at	MG1655	b2134	pbpG
1766576_s_at	MG1655	b0757	galK
1766580_s_at	MG1655	b3473	yhhS
1766581_s_at	CFT073	c2666	NA
1766583_s_at	EDL933	Z0125	aceF
1766589_s_at	MG1655	b0584	fepA
1766591_s_at	MG1655	b1988	nac
1766593_s_at	MG1655	b3033	yqiB
1766616_s_at	MG1655	b3791	wecE
1766619_s_at	MG1655	b3531	NA
1766620_s_at	MG1655	b1660	ydhC
1766621_s_at	MG1655	b0409	secF
1766631_s_at	CFT073	c0526	NA
1766632_s_at	MG1655	b2326	NA
1766633_s_at	MG1655	b3764	yifE
1766635_s_at	CFT073	c3951	NA
1766637_s_at	CFT073	c0590	NA
1766638_s_at	MG1655	b0636	ybeA
1766639_s_at	MG1655	b0598	cstA
1766650_s_at	MG1655	b3917	sbp
1766652_s_at	MG1655	b1628	NA
1766657_s_at	MG1655	b2285	nuoE
1766660_at	CFT073	c5451	NA
1766662_s_at	MG1655	b3610	grxC
1766668_s_at	MG1655	b1047	NA
1766673_s_at	CFT073	c2476	erfK
1766676_s_at	MG1655	b0810	glnP
1766686_s_at	MG1655	b4044	dinF
1766688_s_at	CFT073	c2063	NA
1766690_s_at	MG1655	b0623	cspE
1766699_s_at	CFT073	c4688	NA
1766700_s_at	MG1655	b3149	yraO
1766701_s_at	MG1655	b3072	aer
1766716_s_at	MG1655	b4237	nrdG
1766720_s_at	MG1655	b2680	NA
1766722_s_at	MG1655	b3829	metE
1766723_s_at	EDL933	Z5165	ilvB
1766725_s_at	MG1655	b1446	NA
1766726_s_at	MG1655	b3527	yhjJ
1766731_s_at	MG1655	b2141	yohJ
1766734_s_at	MG1655	b1606	ydgB
1766739_s_at	MG1655	b1205	ychH
1766743_s_at	MG1655	b2811	ygdK
1766745_s_at	MG1655	b4411	ecnB
1766746_s_at	MG1655	b1643	NA
1766747_s_at	MG1655	b1124	potC
1766748_at	CFT073	c4805	NA
1766754_s_at	MG1655	b2711	ygbD
1766761_s_at	MG1655	b2927	epd
1766766_s_at	MG1655	b3349	slyD
1766768_s_at	MG1655	b0001	thrL

1766770_s_at	MG1655	b4149	blc
1766772_s_at	MG1655	b2016	NA
1766774_s_at	MG1655	b3434	yhgN
1766780_s_at	MG1655	b1049	mdoH
1766783_s_at	MG1655	b3838	NA
1766793_s_at	MG1655	b3956	ppc
1766810_s_at	MG1655	b3415	gntT
1766815_s_at	MG1655	b3438	gntR
1766817_s_at	MG1655	b2201	ccmA
1766821_s_at	MG1655	b0153	fhuB
1766832_s_at	MG1655	b4137	cutA
1766834_s_at	MG1655	b1866	aspS
1766836_s_at	MG1655	b3804	hemD
1766837_s_at	MG1655	b3151	yraQ
1766840_s_at	MG1655	b3386	rpe
1766843_s_at	MG1655	b1136	icdA
1766848_s_at	MG1655	b1865	ntpA
1766854_s_at	CFT073	c2047	NA
1766868_s_at	MG1655	b0726	sucA
1766869_s_at	MG1655	b2294	NA
1766870_s_at	CFT073	c3554	NA
1766871_s_at	MG1655	b2011	sbcB
1766873_s_at	MG1655	b3236	mdh
1766876_s_at	MG1655	b2181	yejG
1766877_s_at	MG1655	b1844	NA
1766880_s_at	MG1655	b1180	NA
1766881_s_at	MG1655	b2583	yfiP
1766882_s_at	CFT073	c1683	NA
1766884_s_at	CFT073	c4371	NA
1766885_s_at	CFT073	c1337	NA
1766887_s_at	MG1655	b0852	rimK
1766893_s_at	MG1655	b1837	NA
1766898_s_at	MG1655	b4399	creC
1766899_s_at	MG1655	b1174	minE
1766900_s_at	MG1655	b3065	rpsU
1766901_s_at	MG1655	b1102	fhuE
1766904_s_at	CFT073	c3084	NA
1766907_s_at	CFT073	c4850	yiiL
1766908_s_at	MG1655	b2479	gcvR
1766911_s_at	MG1655	b1708	nlpC
1766918_s_at	MG1655	b1726	NA
1766920_s_at	MG1655	b3925	glpX
1766924_s_at	MG1655	b2158	yeiH
1766925_s_at	MG1655	b0873	NA
1766926_s_at	CFT073	c4960	NA
1766943_s_at	CFT073	c0018	NA
1766945_at	CFT073	c4813	NA
1766948_s_at	CFT073	c2317	NA
1766958_s_at	MG1655	b3990	thiH
1766964_s_at	MG1655	b1603	pntA
1766973_s_at	MG1655	b1177	ycgJ

1766974_s_at	MG1655	b1203	ychF
1766977_s_at	MG1655	b1715	pheM
1766986_s_at	MG1655	b0776	bioF
1766990_s_at	MG1655	b1777	NA
1766993_s_at	MG1655	b3844	NA
1766995_s_at	MG1655	b2193	narP
1766996_s_at	CFT073	c2142	NA
1766997_s_at	MG1655	b3724	phoU
1767002_s_at	MG1655	b0762	NA
1767003_s_at	CFT073	c1367	NA
1767010_s_at	CFT073	c2149	NA
1767016_at	CFT073	c4854	NA
1767019_s_at	MG1655	b1904	NA
1767020_s_at	MG1655	b3825	pldB
1767021_s_at	MG1655	b0847	NA
1767028_s_at	MG1655	b4146	yjeK
1767029_s_at	SAKAI	ECs3179	NA
1767033_s_at	MG1655	b0947	NA
1767038_s_at	MG1655	b0048	folA
1767040_s_at	MG1655	b0722	sdhD
1767041_s_at	MG1655	b0790	ybhP
1767044_s_at	MG1655	b3755	yieP
1767055_x_at	MG1655	b4422	rdlB
1767056_s_at	CFT073	c5327	ytfT
1767064_s_at	MG1655	b1521	uxaB
1767071_s_at	MG1655	b1255	yciC
1767072_s_at	MG1655	b2129	yehX
1767087_s_at	MG1655	b1000	cbpA
1767090_s_at	MG1655	b1659	ydhB
1767091_s_at	MG1655	b2136	yohD
1767099_s_at	MG1655	b3554	yiaF
1767107_s_at	CFT073	c1040	NA
1767108_s_at	MG1655	b0055	NA
1767119_s_at	MG1655	b2962	yggX
1767121_s_at	MG1655	b0481	ybaK
1767122_s_at	MG1655	b0436	tig
1767124_s_at	MG1655	b2996	hybA
1767142_s_at	MG1655	b4359	mdoB
1767143_s_at	MG1655	b3847	pepQ
1767152_at	CFT073	c4858	NA
1767158_s_at	MG1655	b4178	yjeB
1767159_s_at	MG1655	b1668	NA
1767162_s_at	MG1655	b3112	NA
1767164_s_at	MG1655	b1704	aroH
1767168_s_at	MG1655	b2291	NA
1767169_s_at	CFT073	c0480	yaiH
1767176_s_at	MG1655	b4021	pepE
1767179_s_at	MG1655	b0238	gpt
1767183_s_at	MG1655	b2998	NA
1767186_s_at	MG1655	b3225	nanA
1767189_s_at	CFT073	c5272	yjfM

1767190_s_at	MG1655	b2956	yggM
1767192_s_at	MG1655	b2797	sdaB
1767205_s_at	MG1655	b3606	yibK
1767207_s_at	EDL933	Z1248	pflB
1767208_s_at	MG1655	b0025	ribF
1767209_s_at	MG1655	b1853	yebK
1767210_s_at	MG1655	b4197	sgaU
1767212_s_at	MG1655	b0858	ybjO
1767219_s_at	MG1655	b4168	yjeE
1767223_s_at	MG1655	b4004	NA
1767225_s_at	MG1655	b2946	yggJ
1767230_s_at	MG1655	b2611	NA
1767231_s_at	MG1655	b3440	yhhX
1767237_s_at	MG1655	b0687	seqA
1767238_s_at	MG1655	b1241	adhE
1767243_s_at	MG1655	b2839	lysR
1767248_s_at	MG1655	b3007	NA
1767250_s_at	MG1655	b3177	folP
1767252_s_at	MG1655	b3188	nlp
1767254_s_at	MG1655	b4117	adiA
1767263_s_at	MG1655	b2779	eno
1767264_s_at	CFT073	c0161	NA
1767267_s_at	MG1655	b0209	yafD
1767268_x_at	CFT073	c2220	NA
1767276_s_at	MG1655	b1134	NA
1767278_s_at	MG1655	b1849	purT
1767287_s_at	CFT073	c3363	NA
1767289_s_at	MG1655	b0059	hepA
1767291_s_at	MG1655	b3321	rpsJ
1767298_s_at	MG1655	b3125	garR
1767299_s_at	MG1655	b2831	mutH
1767303_s_at	EDL933	Z2878	proQ
1767309_s_at	MG1655	b0861	artM
1767310_s_at	MG1655	b3357	crp
1767319_s_at	EDL933	Z0576	acrB
1767321_s_at	MG1655	b3309	rplX
1767322_s_at	MG1655	b0210	yafE
1767323_s_at	MG1655	b3954	yijO
1767324_s_at	MG1655	b3738	atpB
1767327_s_at	CFT073	c4275	NA
1767330_s_at	MG1655	b3459	yhhK
1767343_s_at	MG1655	b2172	yeiQ
1767347_s_at	MG1655	b3911	cpxA
1767352_s_at	MG1655	b2833	NA
1767357_s_at	MG1655	b3480	nikE
1767358_s_at	EDL933	Z3442	rsuA
1767363_s_at	MG1655	b0886	cydC
1767377_s_at	MG1655	b2606	rplS
1767385_s_at	MG1655	b0931	pncB
1767396_s_at	MG1655	b4412	hokC
1767400_s_at	MG1655	b0917	ycaR

1767401_at	CFT073	c5113	phnA
1767404_s_at	MG1655	b3300	prlA
1767410_s_at	CFT073	c0771	fldA
1767417_s_at	MG1655	b4059	ssb
1767422_s_at	MG1655	b0108	ppdD
1767423_s_at	MG1655	b1908	yecA
1767425_s_at	MG1655	b0928	aspC
1767433_s_at	MG1655	b3034	yqiE
1767434_s_at	MG1655	b2304	NA
1767442_s_at	MG1655	b1765	ydjA
1767445_s_at	MG1655	b0813	ybiF
1767449_s_at	MG1655	b2899	NA
1767450_s_at	CFT073	c4650	NA
1767453_at	MG1655	b4449	sraG
1767455_s_at	MG1655	b1131	purB
1767463_s_at	MG1655	b4376	osmY
1767465_s_at	MG1655	b2300	yfcE
1767468_s_at	MG1655	b0850	ybjC
1767479_s_at	MG1655	b1593	NA
1767487_s_at	MG1655	b3361	fic
1767488_s_at	MG1655	b3362	yhfG
1767493_s_at	MG1655	b2514	hisS
1767503_s_at	MG1655	b3871	NA
1767504_s_at	MG1655	b0091	murC
1767506_s_at	MG1655	b0593	entC
1767508_s_at	MG1655	b1814	sdaA
1767509_s_at	MG1655	b3997	hemE
1767513_s_at	MG1655	b2217	rcsB
1767515_s_at	MG1655	b1917	yecC
1767518_s_at	MG1655	b0721	sdhC
1767519_s_at	EDL933	Z2774	NA
1767520_s_at	MG1655	b2466	NA
1767522_s_at	CFT073	c1094	NA
1767524_s_at	MG1655	b4262	yjgQ
1767525_s_at	MG1655	b0383	phoA
1767527_s_at	CFT073	c4833	rbn
1767537_s_at	EDL933	Z1102	NA
1767543_s_at	MG1655	b1106	ycfN
1767545_at	CFT073	c5455	NA
1767550_s_at	MG1655	b2826	ppdA
1767559_s_at	MG1655	b2699	recA
1767560_s_at	MG1655	b1062	pyrC
1767563_s_at	MG1655	b3295	rpoA
1767564_s_at	MG1655	b0235	NA
1767570_s_at	MG1655	b3964	yijD
1767571_s_at	MG1655	b0453	ybaY
1767572_s_at	MG1655	b0845	NA
1767574_s_at	MG1655	b0145	dksA
1767578_s_at	MG1655	b1290	sapF
1767579_s_at	MG1655	b4234	yjgA
1767581_s_at	MG1655	b1253	yciA

1767596_s_at	MG1655	b2184	yejH
1767598_s_at	MG1655	b0885	aat
1767599_s_at	MG1655	b2827	thyA
1767600_s_at	MG1655	b0738	tolR
1767604_s_at	MG1655	b3472	NA
1767618_s_at	MG1655	b1211	prfA
1767624_s_at	MG1655	b3005	exbD
1767626_s_at	MG1655	b0084	ftsI
1767636_s_at	MG1655	b4154	frdA
1767645_s_at	MG1655	b0012	htgA
1767659_s_at	MG1655	b2914	rpiA
1767664_s_at	CFT073	c3943	NA
1767666_s_at	MG1655	b2517	yfgB
1767668_s_at	CFT073	c1702	NA
1767674_s_at	MG1655	b3261	fis
1767680_at	MG1655	b4443	gcvB
1767682_s_at	MG1655	b2781	mazG
1767684_s_at	MG1655	b0638	phpB
1767690_s_at	MG1655	b3942	katG
1767694_s_at	MG1655	b0628	lipA
1767701_s_at	MG1655	b1597	asr
1767703_s_at	MG1655	b1781	NA
1767705_s_at	MG1655	b3193	yrbD
1767711_s_at	MG1655	b1987	cbl
1767712_s_at	MG1655	b2173	yeiR
1767715_s_at	SAKAI	ECs0488	NA
1767716_s_at	MG1655	b3922	yiiS
1767719_s_at	MG1655	b0729	sucD
1767720_s_at	MG1655	b3753	rbsR
1767723_s_at	MG1655	b3740	gidB
1767724_s_at	MG1655	b3638	radC
1767731_s_at	MG1655	b3168	infB
1767734_s_at	MG1655	b3670	ilvN
1767737_s_at	MG1655	b3640	dut
1767738_s_at	MG1655	b0192	cutF
1767749_s_at	MG1655	b1175	minD
1767751_s_at	MG1655	b0819	ybiS
1767756_s_at	MG1655	b3912	cpxR
1767765_s_at	MG1655	b2318	truA
1767766_s_at	MG1655	b1260	trpA
1767767_s_at	MG1655	b1119	ycfX
1767768_s_at	MG1655	b2533	suhB
1767772_s_at	MG1655	b3358	yhfK
1767775_s_at	MG1655	b3803	hemX
1767776_s_at	MG1655	b3413	yhgH
1767782_s_at	MG1655	b3521	yhjC
1767786_s_at	CFT073	c3054	NA
1767792_s_at	MG1655	b0978	appC
1767794_s_at	MG1655	b3848	yigZ
1767795_s_at	MG1655	b0366	tauB
1767800_s_at	MG1655	b3071	yqjI

1767801_s_at	MG1655	b0463	acrA
1767807_s_at	MG1655	b1089	rpmF
1767812_s_at	MG1655	b2342	NA
1767816_s_at	SAKAI	ECs4149	NA
1767819_s_at	MG1655	b3255	accB
1767821_s_at	MG1655	b3308	rplE
1767825_s_at	MG1655	b0814	ompX
1767829_s_at	MG1655	b4388	serB
1767831_s_at	MG1655	b3062	ttdB
1767833_s_at	MG1655	b0979	appB
1767836_s_at	MG1655	b4246	pyrL
1767841_s_at	MG1655	b3115	tdcD
1767848_s_at	MG1655	b2212	alkB
1767852_s_at	MG1655	b4147	efp
1767856_s_at	MG1655	b1815	NA
1767865_s_at	MG1655	b3060	ygiP
1767873_s_at	MG1655	b1879	flhA
1767874_s_at	MG1655	b3703	rpmH
1767877_s_at	MG1655	b3526	kdgK
1767880_s_at	CFT073	c2964	NA
1767883_s_at	MG1655	b2608	NA
1767888_s_at	MG1655	b1541	NA
1767891_s_at	MG1655	b1678	ynhG
1767908_s_at	MG1655	b2418	pdxK
1767911_s_at	MG1655	b1778	yeaA
1767914_s_at	MG1655	b3549	tag
1767917_s_at	MG1655	b1846	yebE
1767920_s_at	MG1655	b3965	trmA
1767921_s_at	MG1655	b0102	yacF
1767928_s_at	MG1655	b3449	ugpQ
1767934_at	CFT073	c4892	metB
1767950_s_at	MG1655	b0408	secD
1767955_s_at	MG1655	b1085	NA
1767958_s_at	CFT073	c3251	NA
1767961_s_at	MG1655	b0605	ahpC
1767967_s_at	MG1655	b3251	mreB
1767971_s_at	MG1655	b4380	yjjI
1767972_s_at	MG1655	b3731	atpC
1767977_s_at	MG1655	b4199	yjfY
1767981_s_at	MG1655	b0929	ompF
1767983_s_at	MG1655	b4244	pyrI
1767988_s_at	MG1655	b0391	yaiE
1767996_s_at	CFT073	c0065	pdxA
1767997_s_at	MG1655	b3035	tolC
1768000_s_at	MG1655	b2679	proX
1768002_s_at	MG1655	b1637	tyrS
1768003_s_at	MG1655	b1969	yedW
1768004_s_at	CFT073	c0569	NA
1768010_s_at	CFT073	c3421	NA
1768012_s_at	MG1655	b3995	NA
1768015_s_at	MG1655	b1854	pykA

1768017_s_at	MG1655	b1096	pabC
1768018_s_at	CFT073	c4078	NA
1768022_s_at	MG1655	b3396	mrcA
1768025_s_at	MG1655	b2697	alaS
1768029_s_at	MG1655	b3253	yhdH
1768033_s_at	MG1655	b3769	ilvM
1768038_s_at	MG1655	b1655	ydhO
1768043_s_at	MG1655	b2500	purN
1768049_s_at	MG1655	b1123	potD
1768051_s_at	MG1655	b0031	dapB
1768060_s_at	CFT073	c1106	NA
1768062_s_at	MG1655	b3206	ptsO
1768065_s_at	MG1655	b1125	potB
1768074_s_at	MG1655	b1635	gst
1768076_s_at	MG1655	b3011	yqhD
1768079_at	MG1655	b4441	tke1
1768081_s_at	MG1655	b3392	yrfA
1768082_s_at	MG1655	b1114	mfd
1768107_s_at	CFT073	c4141	nirB
1768117_s_at	MG1655	b0848	ybjM
1768118_s_at	MG1655	b3810	yigA
1768120_s_at	MG1655	b1074	flgC
1768139_s_at	MG1655	b3784	rfe
1768144_s_at	CFT073	c3104	NA
1768146_s_at	MG1655	b0220	ykfE
1768151_s_at	MG1655	b0197	metQ
1768160_s_at	MG1655	b4243	yjgF
1768164_s_at	MG1655	b2311	ubiX
1768177_s_at	MG1655	b2316	accD
1768181_s_at	MG1655	b3105	yhaJ
1768183_s_at	MG1655	b1825	NA
1768184_s_at	MG1655	b0973	hyaB
1768191_s_at	MG1655	b2950	yggR
1768198_s_at	MG1655	b4402	yjjY
1768203_s_at	CFT073	c2041	NA
1768209_s_at	MG1655	b2209	eco
1768212_s_at	MG1655	b3621	rfaC
1768221_s_at	MG1655	b2942	metK
1768229_s_at	MG1655	b3712	yieE
1768230_s_at	CFT073	c4084	NA
1768232_s_at	MG1655	b1656	sodB
1768249_s_at	CFT073	c4712	NA
1768250_s_at	MG1655	b3494	yhiO
1768255_s_at	CFT073	c3873	NA
1768260_s_at	MG1655	b3551	bisC
1768266_s_at	MG1655	b0675	nagD
1768271_s_at	CFT073	c4067	NA
1768279_s_at	MG1655	b1525	NA
1768294_s_at	MG1655	b2746	NA
1768297_s_at	MG1655	b4220	ytfM
1768304_s_at	MG1655	b1705	ydiE

1768307_s_at	EDL933	Z4411	ygiG
1768314_s_at	MG1655	b1711	btuC
1768330_s_at	CFT073	c2114	NA
1768331_s_at	MG1655	b3257	yhdT
1768334_s_at	EDL933	Z2747	infC
1768336_s_at	CFT073	c1740	NA
1768337_s_at	CFT073	c3113	NA
1768340_s_at	MG1655	b1816	yoaE
1768341_s_at	MG1655	b4139	aspA
1768345_s_at	MG1655	b0735	ybgE
1768348_s_at	MG1655	b3963	yijC
1768349_s_at	MG1655	b0103	yacE
1768352_s_at	MG1655	b1216	chaA
1768358_s_at	MG1655	b0379	NA
1768362_s_at	MG1655	b1499	NA
1768364_s_at	CFT073	c3090	era
1768365_s_at	MG1655	b4161	yjeQ
1768368_s_at	MG1655	b3391	hofQ
1768379_s_at	MG1655	b0133	panC
1768381_s_at	MG1655	b1068	mviM
1768391_s_at	MG1655	b0454	ybaZ
1768393_s_at	MG1655	b0523	purE
1768397_s_at	MG1655	b3311	rpsQ
1768403_at	SAKAI	ECs5564	NA
1768404_s_at	MG1655	b1271	yciK
1768408_s_at	CFT073	c5056	NA
1768415_s_at	MG1655	b4141	yjeH
1768419_s_at	MG1655	b2798	exo
1768421_s_at	MG1655	b3195	yrbF
1768422_s_at	MG1655	b3095	yqjA
1768427_s_at	CFT073	c2723	NA
1768428_s_at	MG1655	b0654	gltJ
1768432_s_at	MG1655	b0592	fepB
1768435_s_at	MG1655	b1305	pspB
1768439_s_at	MG1655	b3348	slyX
1768443_s_at	MG1655	b0525	ppiB
1768444_s_at	MG1655	b2150	mglB
1768446_s_at	MG1655	b2502	ppx
1768447_s_at	CFT073	c2865	pdxB
1768449_s_at	EDL933	Z4018	NA
1768454_s_at	MG1655	b3244	tldD
1768468_s_at	MG1655	b3827	yigM
1768480_s_at	MG1655	b4148	sugE
1768481_s_at	MG1655	b1836	NA
1768490_s_at	MG1655	b0470	dnaX
1768492_s_at	MG1655	b4261	yjgP
1768494_s_at	MG1655	b2891	prfB
1768504_s_at	MG1655	b1265	trpL
1768509_s_at	MG1655	b4403	lasT
1768510_s_at	EDL933	Z5325	NA
1768511_s_at	MG1655	b1767	ansA

1768512_s_at	MG1655	b1959	yedA
1768518_s_at	MG1655	b4143	NA
1768521_s_at	EDL933	Z5625	yjbF
1768522_s_at	MG1655	b1621	malX
1768523_s_at	CFT073	c2777	nrdB
1768530_s_at	MG1655	b0914	msbA
1768532_s_at	MG1655	b4239	treC
1768534_s_at	MG1655	b1064	grxB
1768537_s_at	MG1655	b4396	rob
1768546_at	CFT073	c4689	NA
1768547_s_at	MG1655	b4371	NA
1768552_s_at	MG1655	b0637	ybeB
1768555_s_at	MG1655	b1071	flgM
1768561_s_at	MG1655	b0176	yaeL
1768562_s_at	MG1655	b3941	metF
1768565_s_at	CFT073	c2951	ptsI
1768567_s_at	MG1655	b2218	rcsC
1768573_s_at	MG1655	b1953	NA
1768589_s_at	MG1655	b0784	moaD
1768595_s_at	MG1655	b3299	rpmJ
1768601_s_at	MG1655	b1291	sapD
1768604_s_at	MG1655	b2554	yfhA
1768609_s_at	SAKAI	ECs5364	NA
1768610_s_at	MG1655	b3522	yhjD
1768619_s_at	MG1655	b2561	yfhH
1768624_s_at	MG1655	b2308	hisQ
1768627_s_at	MG1655	b3506	slp
1768629_s_at	MG1655	b1839	NA
1768632_s_at	MG1655	b2299	NA
1768633_s_at	MG1655	b0918	kdsB
1768638_s_at	MG1655	b3705	yidC
1768651_s_at	MG1655	b2155	cirA
1768660_s_at	MG1655	b3230	rpsI
1768672_s_at	MG1655	b0482	ybaP
1768676_s_at	MG1655	b0750	nadA
1768681_s_at	CFT073	c1363	NA
1768682_s_at	SAKAI	ECs4877	NA
1768684_s_at	MG1655	b1817	manX
1768689_s_at	MG1655	b1482	osmC
1768690_s_at	MG1655	b0405	queA
1768696_s_at	MG1655	b3481	NA
1768704_s_at	MG1655	b0198	metI
1768707_s_at	MG1655	b1252	tonB
1768710_s_at	MG1655	b1083	flgL
1768711_s_at	MG1655	b3198	yrbI
1768717_s_at	MG1655	b2014	yeeF
1768721_s_at	CFT073	c2242	yebS
1768727_s_at	CFT073	c0507	NA
1768730_s_at	CFT073	c4374	NA
1768738_s_at	MG1655	b0756	galM
1768742_s_at	MG1655	b2992	hybE

1768743_s_at	MG1655	b3240	yhcP
1768746_s_at	MG1655	b0199	metN
1768747_s_at	MG1655	b1804	rnd
1768748_s_at	MG1655	b3495	uspA
1768749_s_at	MG1655	b3246	yhdR
1768750_s_at	MG1655	b0916	ycaQ
1768753_s_at	CFT073	c2320	NA
1768757_s_at	MG1655	b1723	pfkB
1768758_s_at	MG1655	b3929	menG
1768759_s_at	MG1655	b1609	rstB
1768762_s_at	MG1655	b3794	wecG
1768763_s_at	MG1655	b2309	hisJ
1768764_s_at	CFT073	c3264	NA
1768775_s_at	MG1655	b3751	rbsB
1768785_s_at	SAKAI	ECs3324	NA
1768790_s_at	MG1655	b2800	fucA
1768793_s_at	MG1655	b2073	NA
1768794_s_at	MG1655	b3306	rpsH
1768796_s_at	MG1655	b0840	deoR
1768801_at	CFT073	c4942	NA
1768808_s_at	MG1655	b2665	ygaU
1768811_s_at	CFT073	c0859	NA
1768821_s_at	MG1655	b0441	NA
1768822_s_at	MG1655	b0806	ybiM
1768827_s_at	MG1655	b0889	lrp
1768828_s_at	MG1655	b0975	hyaD
1768832_s_at	MG1655	b0426	yajQ
1768833_s_at	CFT073	c4086	NA
1768835_at	MG1655	b4457	csrC
1768843_s_at	MG1655	b2805	fucR
1768853_s_at	MG1655	b2307	hisM
1768854_s_at	MG1655	b0415	NA
1768856_s_at	CFT073	c0797	NA
1768860_at	CFT073	c5330	NA
1768864_s_at	MG1655	b0686	ybfF
1768866_s_at	MG1655	b1850	eda
1768867_s_at	MG1655	b3298	rpsM
1768873_s_at	MG1655	b0169	rpsB
1768884_s_at	MG1655	b2698	oraA
1768890_s_at	MG1655	b0390	aroM
1768895_s_at	MG1655	b2671	ygaC
1768897_s_at	MG1655	b2713	hydN
1768901_s_at	MG1655	b1091	fabH
1768902_s_at	MG1655	b2830	ygdP
1768908_s_at	EDL933	Z3376	yehV
1768913_s_at	MG1655	b0010	yaaH
1768917_s_at	MG1655	b3026	ygiY
1768919_s_at	MG1655	b2796	sdaC
1768921_s_at	SAKAI	ECs3305	NA
1768942_s_at	MG1655	b0471	ybaB
1768947_s_at	MG1655	b1220	ychP

1768948_s_at	MG1655	b0459	NA
1768951_s_at	MG1655	b2328	mepA
1768953_s_at	MG1655	b0980	appA
1768954_s_at	MG1655	b0927	ycbL
1768956_s_at	MG1655	b1608	rstA
1768976_s_at	CFT073	c4048	fmt
1768978_s_at	CFT073	c0583	NA
1768980_s_at	CFT073	c3865	NA
1768981_s_at	MG1655	b0385	yaiC
1768984_s_at	MG1655	b0413	ybaD
1768989_s_at	MG1655	b1280	yciM
1769005_s_at	CFT073	c2256	ptrB
1769010_s_at	MG1655	b4030	yjbA
1769012_s_at	CFT073	c0567	NA
1769013_s_at	MG1655	b0893	serS
1769015_s_at	MG1655	b3866	yihI
1769019_s_at	MG1655	b0015	dnaJ
1769020_s_at	MG1655	b0179	lpxD
1769028_s_at	MG1655	b0829	NA
1769031_s_at	MG1655	b1787	yeaK
1769042_s_at	MG1655	b0728	sucC
1769050_s_at	CFT073	c4140	NA
1769054_s_at	MG1655	b1065	yceL
1769056_s_at	MG1655	b1212	hemK
1769057_s_at	CFT073	c2705	NA
1769059_s_at	MG1655	b1585	ynfC
1769060_s_at	MG1655	b3687	ibpA
1769064_s_at	MG1655	b1067	yceH
1769077_s_at	MG1655	b2682	NA
1769079_s_at	CFT073	c1752	NA
1769090_s_at	MG1655	b0128	yadH
1769097_at	MG1655	b4430	rydB
1769101_s_at	MG1655	b1304	pspA
1769103_s_at	EDL933	Z3770	NA
1769105_s_at	CFT073	c3820	NA
1769110_s_at	CFT073	c4069	NA
1769115_s_at	MG1655	b1093	fabG
1769125_s_at	MG1655	b0024	NA
1769146_s_at	MG1655	b1087	yceF
1769148_s_at	MG1655	b0051	ksgA
1769150_s_at	MG1655	b2898	ygfZ
1769151_s_at	MG1655	b0163	yaeH
1769152_s_at	MG1655	b2685	emrA
1769158_s_at	MG1655	b3107	yhaL
1769159_s_at	MG1655	b0842	cmr
1769169_s_at	MG1655	b4354	yjiY
1769171_s_at	MG1655	b2696	csrA
1769178_s_at	MG1655	b2674	nrdI
1769182_s_at	MG1655	b0696	kdpC
1769191_s_at	CFT073	c1078	ycbN
1769192_s_at	MG1655	b1782	NA

1769202_s_at	CFT073	c1294	NA
1769213_s_at	MG1655	b2315	folC
1769218_s_at	CFT073	c3935	NA
1769223_s_at	MG1655	b2818	argA
1769242_s_at	MG1655	b2803	fucK
1769243_s_at	MG1655	b3287	def
1769249_x_at	EDL933	Z1987	NA
1769253_s_at	MG1655	b1662	NA
1769254_s_at	MG1655	b1751	ydjY
1769260_s_at	MG1655	b0724	sdhB
1769267_s_at	MG1655	b1706	NA
1769273_s_at	MG1655	b3178	hflB
1769282_s_at	MG1655	b0460	hha
