# Supplementary material for: Investigating the global genomic diversity of Escherichia coli using a multi-genome DNA microarray platform with novel gene prediction strategies
Source: BMC Genomics. 2011 Jul 6;12:349. doi: 10.1186/1471-2164-12-349 (PMC3146454; doi:10.1186/1471-2164-12-349)
Supplement: Additional File 5 — Conserved Intergenic Regions. Using the MAS 5.0 gene detection method, we filtered those probe sets that were annotated as "intergenic" and called "present" in all 207 isolates. The 232 conserved intergenic probe sets are listed here along with their genome position and length. [file 1471-2164-12-349-S5.PDF]

Additional File 5

ProbeSet ID	MG1655 Start	MG1655 End	Length
1762523_s_at	7960	8237	278
1760454_s_at	11787	12162	376
1767431_s_at	57110	57363	254
1759151_s_at	70049	70386	338
1764772_s_at	83709	84190	482
1765176_s_at	89033	89633	601
1763128_s_at	113220	113443	224
1767687_s_at	131261	131614	354
1764201_s_at	174883	175106	224
1762183_s_at	223409	223770	362
1767421_s_at	230882	231121	240
1768040_s_at	253162	253466	305
1764461_s_at	255717	255976	260
1761272_s_at	395512	395862	351
1768670_s_at	400148	400609	462
1759658_s_at	406395	406651	257
1765115_s_at	418409	418814	406
1760317_s_at	431238	431535	298
1763637_s_at	454014	454356	343
1765118_s_at	455656	455900	245
1767970_s_at	474386	474602	217
1767788_s_at	479933	480477	545
1760974_s_at	502463	502699	237
1764938_s_at	503921	504137	217
1760683_s_at	510604	510864	261
1766522_s_at	531446	531674	229
1767940_s_at	553661	553833	173
1760269_s_at	611718	612037	320
1760865_s_at	613163	613379	217
1759381_s_at	631223	631611	389
1764440_s_at	637857	638167	311
1767727_s_at	668152	668518	367
1764784_s_at	674007	674240	234
1767128_s_at	696357	696735	379
1762723_s_at	702835	703166	332
1762590_s_at	706981	707556	576
1768541_s_at	709870	710157	288
1766909_s_at	727956	728356	401
1763618_s_at	741780	742049	270
1762668_s_at	753692	754399	708
1768658_s_at	779613	779776	164
1760830_s_at	784541	784855	315
1765619_s_at	815871	816266	396
1768936_s_at	829879	830094	216
1760602_s_at	836660	836887	228
1765223_s_at	840755	841018	264
1759245_s_at	847228	847630	403
1759852_s_at	848135	848432	298
1768430_s_at	849321	849672	352
1765069_s_at	879710	879949	240
1768906_s_at	882612	882895	284
1760446_s_at	889043	889311	269
1767497_s_at	892657	893006	350
1761254_s_at	902958	903174	217
1764692_s_at	921814	922135	322
1763876_s_at	925667	925950	284
1759433_s_at	931274	931817	544
1768754_s_at	939944	940268	325

1765455_s_at	953690	954094	405
1761292_s_at	980010	980269	260
1765759_s_at	986206	986807	602
1768606_s_at	989580	989844	265
1765145_s_at	1014683	1014937	255
1760422_s_at	1019277	1019632	356
1766124_s_at	1020143	1020360	218
1760551_s_at	1030936	1031361	426
1759859_s_at	1050399	1050683	285
1762213_s_at	1062999	1063258	260
1765768_s_at	1073235	1073464	230
1762016_s_at	1108165	1108557	393
1764913_s_at	1115806	1116029	224
1761033_s_at	1120179	1120464	286
1764615_s_at	1150628	1150837	210
1759117_s_at	1156798	1157091	294
1766198_s_at	1160775	1161107	333
1768452_s_at	1184818	1185066	249
1759948_s_at	1225304	1225822	519
1762118_s_at	1233941	1234160	220
1759577_s_at	1236465	1236793	329
1759502_s_at	1241169	1241388	220
1764685_s_at	1257737	1258013	277
1761611_s_at	1272823	1273147	325
1764546_s_at	1276842	1277179	338
1769230_s_at	1278572	1279086	515
1760341_s_at	1292146	1292749	604
1762102_s_at	1297345	1297820	476
1759110_s_at	1298469	1299205	737
1764282_s_at	1306670	1307039	370
1767692_s_at	1327137	1327355	219
1765068_s_at	1328693	1329071	379
1768057_s_at	1331670	1331878	209
1765548_s_at	1332854	1333854	1001
1765961_s_at	1341353	1341620	268
1759623_s_at	1342371	1342780	410
1768692_s_at	1395117	1395388	272
1769272_s_at	1545194	1545424	231
1765037_s_at	1620544	1620983	440
1759311_s_at	1668977	1669372	396
1763380_s_at	1669709	1669983	275
1763381_s_at	1671526	1671936	411
1767257_s_at	1675928	1676450	523
1765964_s_at	1702701	1702972	272
1763856_s_at	1710183	1710792	610
1764919_s_at	1717627	1717899	273
1765876_s_at	1732126	1732458	333
1769127_s_at	1735315	1735867	553
1763050_s_at	1739147	1739436	290
1764277_s_at	1741267	1741480	214
1762433_s_at	1744152	1744458	307
1768543_s_at	1753166	1753721	556
1759667_s_at	1755135	1755444	310
1765705_s_at	1762411	1762736	326
1766039_s_at	1785137	1785468	332
1760615_s_at	1790045	1790290	246
1761674_s_at	1800595	1801117	523
1763793_s_at	1821310	1821538	229
1761719_s_at	1823650	1823978	329

1759197_s_at	1830007	1830451	445
1765701_s_at	1860454	1860794	341
1759679_s_at	1876765	1877030	266
1759967_s_at	1899610	1900071	462
1762270_s_at	1903284	1903657	374
1761902_s_at	1927732	1928057	326
1760503_s_at	1932629	1932862	234
1762981_s_at	1934339	1934675	337
1768329_s_at	1948547	1948822	276
1760120_s_at	1956985	1957303	319
1759919_s_at	1957877	1958085	209
1764920_s_at	2059956	2060281	326
1762732_s_at	2085091	2085350	260
1769052_s_at	2141025	2141287	263
1768829_s_at	2165052	2165133	82
1761395_s_at	2212665	2212885	221
1765349_s_at	2238369	2238647	279
1760625_s_at	2241673	2241929	257
1760520_s_at	2244790	2245082	293
1765444_s_at	2261516	2261882	367
1763440_s_at	2264043	2264264	222
1768765_s_at	2267588	2267998	411
1767460_s_at	2276258	2276589	332
1760657_s_at	2288102	2288519	418
1763595_s_at	2301518	2301924	407
1769160_s_at	2310770	2311103	334
1759341_s_at	2311197	2311507	311
1763377_s_at	2350395	2350666	272
1759570_s_at	2355824	2356061	238
1769073_s_at	2378439	2378741	303
1760155_s_at	2404662	2405580	919
1765143_s_at	2411153	2411489	337
1761792_s_at	2424809	2425028	220
1765128_s_at	2428784	2429041	258
1766085_s_at	2438141	2438404	264
1759680_s_at	2458979	2459319	341
1768420_s_at	2463028	2463320	293
1760013_s_at	2493313	2493598	286
1761863_s_at	2494586	2495076	491
1762918_s_at	2510727	2511061	335
1767916_s_at	2516252	2516471	220
1768242_s_at	2518693	2518950	258
1765018_s_at	2525965	2526180	216
1759584_s_at	2529254	2529482	229
1763088_s_at	2531401	2531783	383
1764313_s_at	2539274	2539698	425
1760075_s_at	2576398	2576685	288
1766122_s_at	2588729	2589266	538
1762876_s_at	2590755	2591091	337
1764584_s_at	2595639	2595850	212
1761282_s_at	2613902	2614113	212
1767760_s_at	2618920	2619216	297
1762207_s_at	2640865	2641148	284
1763067_s_at	2683528	2683854	327
1759908_s_at	2693564	2693958	395
1769086_s_at	2702084	2702354	271
1759848_s_at	2708033	2708439	407
1769082_s_at	2729179	2729619	441
1763480_s_at	2735516	2735618	103

1768923_s_at	2748730	2748851	122
1761710_s_at	2751478	2751814	337
1760066_s_at	2796517	2797184	668
1761253_s_at	2816668	2816982	315
1762127_s_at	2817169	2817402	234
1768773_s_at	2874353	2874603	251
1759200_s_at	2889922	2890236	315
1760216_s_at	2907689	2907915	227
1768789_s_at	2922538	2922756	219
1767632_s_at	2925695	2926250	556
1765085_s_at	2967000	2967683	684
1761721_s_at	2974408	2974620	213
1764421_s_at	3030836	3031084	249
1766961_s_at	3039091	3039332	242
1766672_s_at	3054986	3055197	212
1759190_s_at	3067828	3068184	357
1762913_s_at	3154533	3154753	221
1768031_s_at	3161498	3161730	233
1762108_s_at	3182483	3182795	313
1761211_s_at	3198607	3198847	241
1759964_s_at	3208185	3208421	237
1762839_s_at	3216718	3217023	306
1763814_s_at	3245069	3245412	344
1764372_s_at	3315931	3316277	347
1767242_s_at	3320147	3320373	227
1765294_s_at	3326342	3326603	262
1763123_s_at	3331093	3331350	258
1760632_s_at	3334460	3334603	144
1762897_s_at	3351686	3352266	581
1763962_s_at	3376287	3376504	218
1768281_s_at	3381904	3382337	434
1763135_s_at	3389664	3390093	430
1760644_s_at	3398785	3399028	244
1760862_s_at	3402255	3403072	818
1768806_s_at	3407589	3407916	328
1761366_s_at	3475057	3475276	220
1768357_s_at	3476135	3476438	304
1761056_s_at	3483456	3483756	301
1763272_s_at	3489935	3490204	270
1765810_s_at	3523788	3524106	319
1760541_s_at	3530078	3530455	378
1763805_s_at	3550107	3550717	611
1762167_s_at	3571136	3571407	272
1762582_s_at	3575186	3575415	230
1765131_s_at	3595192	3595614	423
1763808_s_at	3598415	3598658	244
1765856_s_at	3606628	3606847	220
1764058_s_at	3635041	3635271	231
1759480_s_at	3637351	3637740	390
1760566_s_at	3638176	3638491	316
1767709_s_at	3672006	3672415	410
1767615_s_at	3679571	3679790	220
1767061_s_at	3697829	3698191	363
1765833_s_at	3703420	3703726	307
1766099_s_at	3716794	3717106	313
1764914_s_at	3717398	3717677	280
